# Future distribution of the epiphytic leafless orchid (*Dendrophylax lindenii*), its pollinators and phorophytes evaluated using niche modelling and three different climate change projections

**DOI:** 10.1038/s41598-023-42573-5

**Published:** 2023-09-14

**Authors:** Marta Kolanowska

**Affiliations:** Faculty of Biology and Environmental Protection, Department of Geobotany and Plant Ecology, University of Lodz, Banacha 12/16, 90-237 Lodz, Poland

**Keywords:** Ecology, Climate-change ecology, Ecological modelling

## Abstract

The identification of future refugia for endangered species from the effects of global warming is crucial for improving their conservation. Because climate-driven shifts in ranges and local extinctions can result in a spatial mismatch with their symbiotic organisms, however, it is important to incorporate in niche modelling the ecological partners of the species studied. The aim of this study was to evaluate the effect of climate change on the distribution of suitable niches for the ghost orchid (*Dendrophylax lindenii*) and its phorophytes and pollinators. Thus, its five species of host trees and three pollen vectors were included in the analysis. Climatic preferences of all the species studied were evaluated. The modelling was based on three different climate change projections and four Shared Socio-economic Pathway trajectories. All the species analysed are characterized by narrow temperature tolerances, which with global warming are likely to result in local extinctions and range shifts. *D. lindenii* is likely to be subjected to a significant loss of suitable niches, but within a reduced geographical range, both host trees and pollen vectors will be available in the future. Future conservation of this orchid should focus on areas that are likely be suitable for it and its ecological partners.

## Introduction

Climate change and subsequent environmental challenges are urgent global issues^1^. While scientists generally agree that the accumulation of greenhouse gases is mainly due to human activities^2^, the consequences of global warming are still being discussed^3–6^. The most obvious outcome of climate change is a reduction in biodiversity due to alterations in local climatic conditions^7–10^. Many studies evaluate the future availability of suitable niches for different organisms occurring in various habitats^11–15^. Generally, three different directions of change in range are expected to occur due to climate change: (1) expansion^16–18^, (2) shift (spatial or altitudinal)^19–21^ or (3) contraction^22,23^. Alterations in range could potentially reshape ecological communities and modify ecosystem function and ecosystem services^24^. Species that are able to expand their distribution are highly likely to survive, but species in the recipient community are exposed to numerous threats^25^. Spatial expansions can result in allele frequency gradients, promote the migration of rare variants into newly occupied areas, induce the restructuring of newly colonized regions into distinct sectors of low genetic diversity, or lead to introgressive hybridization of local genes and the genomes of the invading species^26^. As recently revealed, species that actively migrate towards refuges can preserve higher levels of diversity there if the contraction is rapid, whereas if it is slow it is likely the diversity will be low^25^. Obviously, large-scale range contractions deplete populations and decrease genetic diversity by altering the spatial configuration and the area of suitable habitat^27^ and increase the risk of species going extinct^28^.

Climate-driven species extinctions are the most apparent consequences of global warming, but little is known about how it modifies ecological interactions, which can also affect populations and result in extinctions^29^. According to the most recent Assessment of climate change, published in August 2021 by the United Nations (UN) Intergovernmental Panel on Climate Change (IPCC), five different scenarios of socioeconomic global changes up to 2100 are considered^30^. In SSP1-1.9 scenario very low greenhouse gas (GHG) emissions is predicted with CO_2_ emissions cut to net zero around 2050. Similarly the SSP1-2.6 scenario forecasts CO_2_ emissions cut to net zero around 2075^30^. The SSP2-4.5 estimate is based on intermediate GHG emissions with CO_2_ emissions around current levels until 2050, then falling but not reaching net zero by 2100^30^. SSP3-7.0 and SSP5-8.5 predict high and very high GHG emissions with CO_2_ emissions doubling by 2100 and tripling by 2075, respectively^30^. Estimated warming (2081–2100) in these scenarios range from 1.4 °C for SSP1-1.9 to 4.4 °C for SSP5-8.5. Predicted future warming will increase the rate of extreme weather events (heatwaves, heavy precipitation and droughts)^31^. With the warming of the Earth’s surface it is predicted evaporation will increase, resulting in an increase in overall precipitation^32^. However, shifts in wind patterns and ocean currents will also cause some areas to experience reduced precipitation^33^. While changes in both temperature and precipitation will differ spatially in the world all predicted climate change scenarios will affect the structure of ecological networks, which is crucial for coexistence and stability of species^34^. Without doubt, epiphytic vascular plants are an important part of the tropical and subtropical flora affecting ecosystem functioning and local biological diversity^35^. So far little is known about the potential effect of global warming on epiphytes characterized by specific phorophyte preferences and specialist pollinators. In this context, tropical orchids are recognized as flagship examples of complex relationships between plant, host tree and pollen vector^36^.

In this study *Dendrophylax lindenii* (commonly known as the ghost orchid), one of the most intensively studied epiphytic orchid was chosen as the model species for analysing the effect of global warming on highly specialized plants with specific phorophytes and pollinators. About 70% of the more than 27,000 orchids are epiphytic herbaceous plants^36^, but the pollinators of most of them are unknown^37,38^. In addition, little is known about their phorophyte associations. The ghost orchid is probably the only epiphytic orchid for which its pollinators^39,40^, fragrance composition^41^ and host tree affinities^42^ have been studied. Leafless *D. lindenii* is native to lowland forests in Florida and Cuba^42^. It was also reported from Bahamas^43^, but this information is not confirmed^42,44^. For a long time the giant sphinx (*Cocytius antaeus*, Sphingidae) was considered to be the sole pollinator of *Dendrophylax lindenii* as its proboscis is of comparable length to the corolla of *D. lindenii*^45^. Using remotely controlled cameras Houlihan et al.^39^ revealed that it is also pollinated by the fig sphinx moth (*Pachylia ficus*, Sphingidae) and pawpaw sphinx moth (*Dolba hyloeus*, Sphingidae) in Florida^39,40^. The size and bark characteristics of the host (phorophyte) are important factor affecting the distribution of epiphytes. Populations of *D. lindenii* in Southern Florida and Cuba are separated by about 600 km and occur in different habitats^45^. It is therefore not surprising that in its geographical range it occurs on different species of trees. *D. lindenii* grows only on swamp ash (*Fraxinus caroliniana*, Lamiales) and pond apple (*Annona glabra*, Magnoliales) in Florida^45^. The host diversity in Cuba is larger as it is recorded on 18 species of trees, but mainly on boa wood persimmon (*Diospyros crassinervis*, Ericales), swamp redwood (*Erythroxylum areolatum*, Malpighiales) and toothed maiden plum (*Comocladia dentata*, Sapindales)^45^.

Ecological niche modelling (ENM) was used in this study to evaluate the future distribution of suitable niches for *D. lindenii*, its phorophytes and pollinators. Based on the results changes in the overlap between this orchid and its ecological partners was assessed.

## Methods

### List of localities

The database of the localities of ghost orchid*,* its pollinators and phorophytes was compiled based on herbarium data and the public database GBIF. No plants were collected in this study. Five preferred phorophytes were included in the analyses (Table 1). While pollen is known to be transferred by two hawkmoths, the modelling also included *Cocytius antaeus,* which visits flowers of *D. lindeni* despite the fact that no pollinia removal is recorded for this moth^39^.

**Table 1:** Species included in modelling with corresponding GBIF datasets and number of records used in the analyses after spatial thinning.

Species	GBIF data	Records used in ENM
*Dendrophylax lindenii*	https://www.gbif.org/occurrence/download/0255545-220831081235567	23
*Dolba hyloeus*	https://www.gbif.org/occurrence/download/0265829-220831081235567	122
*Cocytius antaeus*	https://www.gbif.org/occurrence/download/0106057-210914110416597	64
*Pachylia ficus*	https://www.gbif.org/occurrence/download/0255546-220831081235567	234
*Annona glabra*	https://www.gbif.org/occurrence/download/0265815-220831081235567	343
*Comocladia dentata*	https://www.gbif.org/occurrence/download/0265816-220831081235567	26
*Diospyros crassinervis*	https://www.gbif.org/occurrence/download/0265817-220831081235567	77
*Erythroxylum areolatum*	https://www.gbif.org/occurrence/download/0265818-220831081235567	74
*Fraxinus caroliniana*	https://www.gbif.org/occurrence/download/0265820-220831081235567	73

Because the quality of input data is crucial element of the ENM model’s accuracy^46,47^, only localities which could be georeferenced with a precision of at least 1 km were used in ENM analyses and all other records were removed as they could not be assigned to the specific grid cell within study area. Since previous analyses indicated that MaxEnt performs well with small sample sizes^48,49^, the database was further filtered to reduce the effect of uneven, or biased, species occurence collections on spatial model outcomes^50,51^. The spatial thinning was done using SDMtoolbox 2.3 for ArcGIS^52,53^. The location data were spatially filtered at 5 km^2^ to maximize the number of spatially independent localities (Table 1, Supplementary Annex 1).

### Climatic niche modelling

The modelling of the current and future distributions of the species studied was done using the maximum entropy method implemented in MaxEnt version 3.3.2^54–56^, which is based on presence-only observations. Gridded bioclimatic variables (“bioclims”) representing yearly (annual mean temperature, annual precipitation, annual range in temperature and precipitation), seasonal (temperature in the coldest and warmest months, precipitation in the wettest and driest quarters) and monthly means and extremes in temperature and precipitation were used^57^. These environmental predictors are derived from monthly temperature and rainfall values^57^. Bioclims in 30 arc-seconds of interpolated climate surface downloaded from WorldClim v. 2.1 were used for the modelling^58^. To avoid problems associated with auto-correlation 12 of 19 variables were removed due to the high correlations between them (> 0.9) as indicated by Pearsons’ correlation coefficient computed using SDMtoolbox 2.3 for ArcGIS^52,53^ (Supplementary Annex 2). Of the two remaining variables the one representing the original input climate data and not derived from several layers or a subset of the data^52,53^ was kept for modelling. The final list of bioclimatic variables used in the analyses is provided in Table 2. The area included in the modelling of suitable niches of *D. lindenii* was restricted to 6.15–30.92° N and 57.74–113.82° W due to limited geographical range of this species. The area included in the analyses of the more broadly distributed phorophytes and pollinators was larger, 16.86–30.73° N and 63.88–88.40° W.

**Table 2:** Bioclimatic variables.

Code	Description	ENM
bio1	Annual mean temperature	+
bio2	Mean diurnal range [mean of monthly (max temp—min temp)]	+
bio3	Isothermality (bio2/bio7) (× 100)	
bio4	Temperature seasonality (standard deviation × 100)	+
bio5	Max temperature in warmest month	
bio6	Min temperature in coldest month	+
bio7	Temperature annual range (bio5-bio6)	
bio8	Mean temperature in wettest quarter	
bio9	Mean temperature in driest quarter	
bio10	Mean temperature in warmest quarter	
bio11	Mean temperature in coldest quarter	
bio12	Annual precipitation	+
bio13	Precipitation in wettest month	
bio14	Precipitation in driest month	+
bio15	Precipitation seasonality (coefficient of variation)	
bio16	Precipitation in wettest quarter	
bio17	Precipitation in driest quarter	
bio18	Precipitation in warmest quarter	
bio19	Precipitation in coldest quarter	+

Layers used in ENM analyses marked with ‘ + ”.

Predictions of the future extent of the climatic niches of the species studied in 2080–2100 were made based on four projections for four Shared Socio-economic Pathways (SSPs): 1–2.6, 2–4.5, 3–7.0 and 5–8.5^59–61^. SSPs are trajectories adopted by the Intergovernmental Panel on Climate Change (IPCC), comprising narrative descriptions of future world development^62^. SSP storylines describe contrasting visions of future society and the assumed climate change challenges, with global warming in 2100 ranging from a low of 3.1 °C to a high of 5.1 °C above pre-industrial levels^63^. Three different simulations of future climate developed by the Coupled Model Intercomparison Project Phase 6 (CNRM), Goddard Institute for Space Studies (GISS) and Institute for Numerical Mathematics (INM) were used. These projections were chosen as they predict the biggest differences in the maximum temperature and precipitation within the area studied (Supplementary Annex 3).

In all analyses the maximum number of iterations was set at 10,000 and convergence threshold to 0.00001. A neutral (= 1) regularization multiplier value and auto features were used. The “random seed” option provided a random test partition and background subset for each run and 30% of the samples were used as test points. The run was performed as a bootstrap with 100 replicates. The output was set to logistic. The “fade by clamping” function in MaxEnt was used to prevent extrapolations outside the environmental range of the training data^64^. All analyses of GIS data were carried out using ArcGis 10.6 (Esri, Redlands, CA, USA). The evaluation of the models was done using the most common metric, the area under the ROC curve (AUC)^50,65^.

SDMtoolbox 2.3 for ArcGIS^52,53^ was used to visualize changes in the distribution of suitable niches for the orchid studied and its pollinator due to global warming. To compare the distribution created for current climatic conditions with future predictions all SDMs were converted into binary rasters and projected using the Goode homolosine as a projection. The presence thresholds used in the analyses equalled the calculated minimum training presence threshold^66^. Predicted niche occupancy (PNO), which integrates species probability distributions (derived using MaxEnt) with respect to climatic variables, was used to visualize climatic preferences of all the species studied^67^.

## Results

### Model evaluation and limiting factors

**Figure 1:**
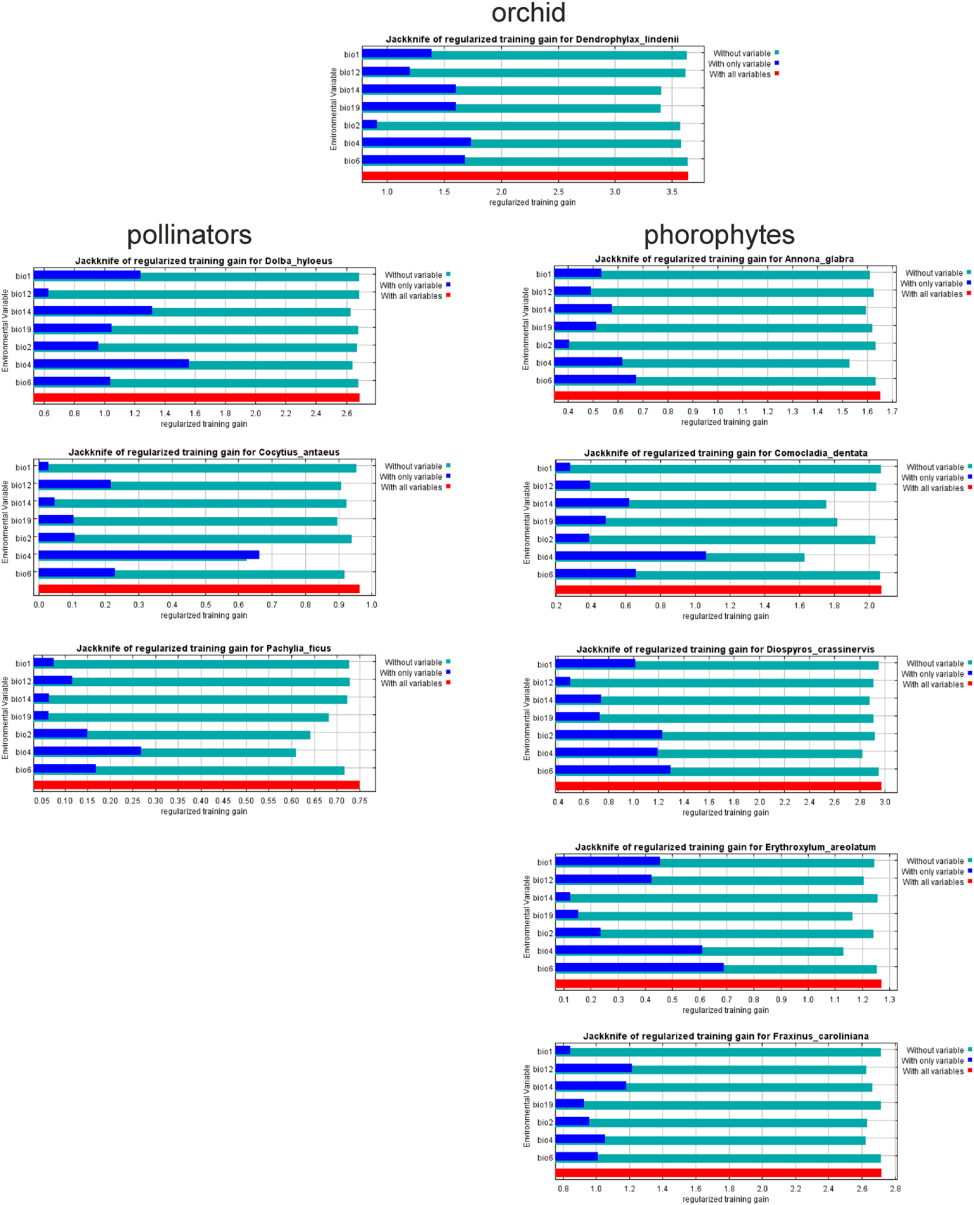
Results of the jack-knife tests of the importance of the variables. Graphs compiled in MaxEnt.

The performance indices of the different models are presented in Table 3. Generally, all models had high AUC values (0.851–0.996) indicating the analyses are reliable. According to the results of the jack-knife tests (Fig. 1) the *D. lindenii* environmental variable with highest gain when used in isolation was bio4, which therefore was the most useful piece of information. The environmental variable that decreased the gain the most when omitted was bio19, which therefore appears to include information that is not present in the other variables.

**Table 3:** Results of the evaluations of the models and values used as presence thresholds (SD is standard deviation).

Species	AUC	Minimum training presence threshold
*Dendrophylax lindenii*	0.996 (SD = 0.002)	0.3103
*Dolba hyloeus*	0.978 (SD = 0.002)	0.0887
*Cocytius antaeus*	0.897 (SD = 0.017)	0.1058
*Pachylia ficus*	0.851 (SD = 0.015)	0.0491
*Annona glabra*	0.935 (SD = 0.006)	0.0057
*Comocladia dentata*	0.981 (SD = 0.004)	0.3251
*Diospyros crassinervis*	0.984 (SD = 0.003)	0.0441
*Erythroxylum areolatum*	0.928 (SD = 0.011)	0.1128
*Fraxinus caroliniana*	0.984 (SD = 0.003)	0.1134

For orchid pollinators, bio4 was the most useful piece of information. The same variable included more information than the other variables in models for *Cocytius antaeus* and *Pachylia ficus*. For *Dolba hyloeus* the environmental variable that decreased the gain the most when omitted was bio14.

Similarly, for orchid phorophytes bio4 was the most useful piece of information. Except for *Fraxinus caroliniana* for which bio12 included the most useful information, distribution of all phorophytes was associated with bio6, which was the variable with highest gain when used in isolation.

The PNO profiles (Supplementary Annex 4) indicated that most species have two optimum annual mean temperatures (bio1): 19–21 °C and 24–25 (26) °C. The temperature seasonality, which was an important feature of the models of most of the taxa analysed for the different species. For *D. lindenii* it was 300–500, *Dolba hyloeus* 600–800, *Cocytius antaeus* 100–300 and *Pachylia ficus* 20–400. The host trees of the orchids also differ in their tolerance of bio4, with areas with suitable scores for occurrence for *Annona glabra* 100–400, *Comocladia dentata* 50–300, *Diospyros crassinervis* 100–700, *Erythroxylum areolatum* 150–500 and *Fraxinus caroliniana* 300–850. These species are tolerant of variation in annual precipitation. Ghost orchid grows in areas characterized by 500–2500 mm of rain and similar values (700–2300 mm) are preferred by *Cocytius antaeus. Dolba hyloeus* and *Pachylia ficus* are associated with lower rainfalls (20–1000 mm and 50–7000 mm, respectively). Similarly, rather broad tolerance of the amount of annual rainfall is reported for other orchid phorophytes. *Comocladia dentata* and *Diospyros crassinervis* can grow in dry areas, whereas *Annona glabra*, *Erythroxylum areolatum* and *Fraxinus caroliniana* require at least 800 mm of rain per year. Generally, all the species studied are tolerant of low precipitation in the driest month (bio14), with most requiring as little as 25–50 mm of rain in the dry season. Only *Pachylia ficus* and *Annona glabra* did not occur in regions with values of bio4 lower than 100. Most species (except *Annona glabra* and *Fraxinus caroliniana*) can survive little rain in the coldest month of the year (bio19).

### Potential future distribution of *Dendrophylax lindenii*, its pollinators and phorophytes

**Figure 2:**
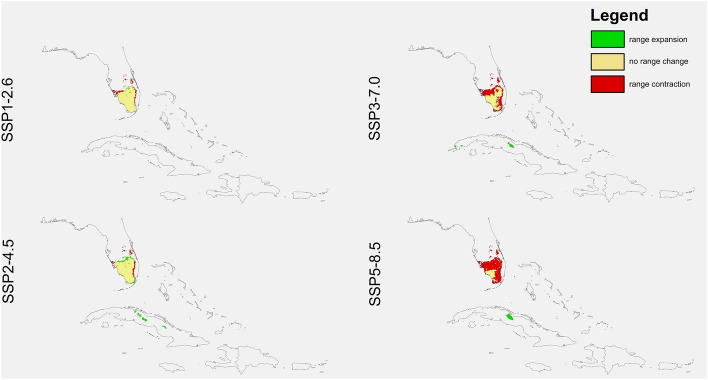
Predicted changes in the distribution of *D. lindenii* in Florida and Cuba in the various climate change scenarios according to CNRM preojections*.* Maps created in ArcGIS based on MaxEnt results.

**Figure 3:**
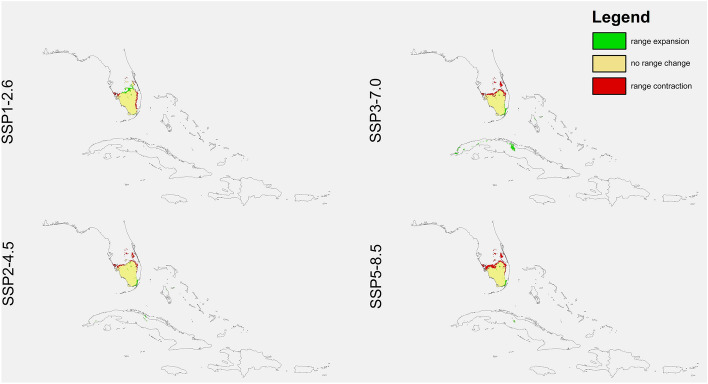
Predicted changes in the distribution of *D. lindenii* in Florida and Cuba in the various climate change scenarios according to GISS preojections*.* Maps created in ArcGIS based on MaxEnt results.

**Figure 4:**
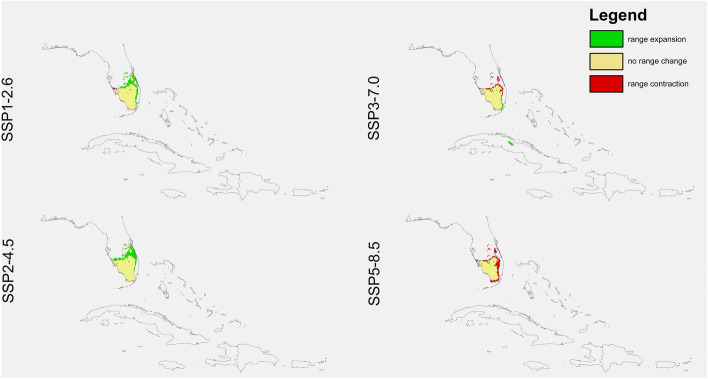
Predicted changes in the distribution of *D. lindenii* in Florida and Cuba in the various climate change scenarios INM preojections*.* Maps created in ArcGIS based on MaxEnt results.

Habitats suitable for *D. lindenii* declined in all simulations of the scenarios SSP3-7.0 (4.4–41%) and SSP5-8.5 (18.1–71.6%) (Figs. 2, 3 and 4, Table 4). However, using the two less severe global warming scenarios resulted in different predictions. In CNRM it was predicted that the number of suitable niches for this species will be less in SSP1-2.6, but it’s potential range in SSP2-4.5 will be greater. The GISS simulation was the most pessimistic as according to this analysis all climate change scenarios will result in a loss of niches for *D. lindenii*. The INM was the most optimistic with a predicted niche expansion in both SSP1-2.6 and SSP2-4.5 (24.7%, 29.9%). Generally, this orchid will lose niches in the north-eastern part of its range. Its range in Florida will contract, while that in Cuba will increase slightly.

**Table 4:** Changes in the coverage (km^2^) of suitable niches for *D. lindenii.*

Species	Projection	Scenario	Range expansion	No change in range	Range contraction	Change
*Dendrophylax lindenii*	CNRM	SSP1-2.6	353.0521	24,941.54	3893.069	− 12.28%
		SSP2-4.5	3912.923	25,914.37	2920.233	+ 3.44%
		SSP3-7.0	1509.751	15,414.31	13,420.3	− 41.31%
		SSP5-8.5	2215.855	5956.136	22,878.47	− 71.66%
	GISS	SSP1-2.6	1729.006	26,092.19	2742.412	− 3.51%
		SSP2-4.5	1168.784	25,413.71	3420.894	− 7.81%
		SSP3-7.0	3157.615	24,397.72	4436.89	− 4.44%
		SSP5-8.5	835.5855	22,771.43	6063.174	− 18.13%
	INM	SSP1-2.6	7608.317	28,354.66	479.9437	+ 24.72%
		SSP2-4.5	8695.959	28,767.28	67.33024	+ 29.92%
		SSP3-7.0	1426.02	24,837.09	3997.517	− 8.92%
		SSP5-8.5	171.7784	21,182.27	7652.34	− 25.94%

Global warming will variously affect the pollinators of *D. lindenii* (Supplementary Annex 5, Supplementary Annex 6)*.* Both GISS and INM projections predict expansion of the range of *Dolba hyloeus* (5–28%), whereas GISS predicted a small loss of part of its currently suitable niches in the SSP3-7.0 and SSP5-8.5 scenarios. On the other hand, all models indicated niche loss for *Cocytius antaeus.* The range contraction was the most extensive in the CNRM projection (3–26%) with the most optimistic the INM simulation (0.2–7% of niche loss). The future of *Pachylia ficus* is not clear. CNRM predictions indicated niche loss in three of the four analysed climate change scenarios (0.5–19%), with all other calculations predicting a minor expansion in the potential range of this species (0.3–4.3%).

Phorophytes of *D. lindenii* respond differently to global warming (Supplementary Annex 5, Supplementary Annex 7). The potential range of *Annona glabra* should not change significantly. The most pessimistic CNRM projection for the SSP5-8.5 scenario indicated 4.5% loss of currently suitable niches, while the most favourable INM simulation for the SSP2-4.5 scenario predicted 7.6% range expansion for this species. Analyses of the effects of global warming on *Comocladia dentata* were inconclusive. While CNRM projections indicated a minor but significant range contraction for this species (3.2–26%), both GISS and INM simulations predicted a small expansion in suitable niches (3.2–6.3%). All the projections analysed indicated range contraction for *Diospyros crassinervis* and *Fraxinus caroliniana* with loses, respectively, of 2–52% and 0.6–67% of currently available niches. In contrast, global warming will favour *Erythroxylum areolatum,* which will expand its current potential range by 3–16%.

### Future availability of orchid pollinators

Considering the availability of pollen vectors (Supplementary Annex 8, Supplementary Annex 9), currently all regions suitable for *D. lindenii* are within the potential ranges of *P. ficus* and *C. antaeus,* but *D. hyloeus* is restricted to less than 25% of this orchid’s range. The pawpaw sphinx will extend its range in the future becoming more available for *D. lindenii.* The availability of the giant sphinx will be reduced according to the CNRM projection and in the worst-case scenario *D. lindenii* will lose this pollinator in 74% of its current range. Two other projections, GISS and INM, are more optimistic in predicting not more than 15% pollinator loss. The fig sphinx will be available as a pollen vector throughout the geographical range of *D. lindenii* in the future*.*

### Future availability of orchid phorophytes

Considering the overlap of the ranges of the orchid and its phorophytes (Supplementary Annex 8, Supplementary Annex 10), currently *C. dentata* only seems to be an occasional host for *D. lindenii*, but it will become more available for this species in the future. *Diospyros crassinervis,* which is currently present in ca. 1/3 of this orchid’s range becomes less available as a phorophyte in the CNRM and GISS projections, but there is an increase in its overlap with the range of *D. lindenii* in the INM simulation. Three other phorophytes are currently broadly distributed and available for the populations of the orchid studied. *Annona glabra* and *Erythroxylum areolatum* will remain common in all areas suitable for this orchid, whereas *Fraxinus caroliniana* will become less frequent and in the worst-case scenario it will occur in only 0.5% of the range of *D. lindenii*. The predictions of other simulations and climate change scenarios, however, are more optimistic and predict a not more than 33% decrease in overlap for these species.

## Discussion

As a result of global warming there is a growing interest in identifying potential refugia for endangered species^68^. The identification of such areas is crucial for improving the conservation of threatened species like *D. lindenii.* Species distribution models based on presence-only data and mapped climatic variables are widely used for analyzing the distribution of suitable niches for endangered species and are useful tools for biodiversity conservation^69,70^. There are, however, several forecasts and the future situation of climate change is very uncertain. Global climate models (GCMs) rely on information for simulating and projecting future changes in climate based on estimates of changes in mixtures of greenhouse gases, anthropogenic and volcanic aerosols, ozone and solar radiance^71–73^. There are three main sources of uncertainty in climate projections: natural climate variability (e.g. semi-cyclical phenomena), emissions uncertainty (trajectories of emissions are based on a set of assumptions) and model uncertainty. It is generally presumed that estimates based on the results of several models are more reliable than those based on a single model^74^ and in this study three different simulations of the future climate were used to present a broad spectrum of possible changes in the distribution of suitable niches of the species studied.

Any simulation of future temperature and precipitation are unsure, but even more difficult is the evaluation the effect of global warming on particular species because climate change will not only affect local climatic conditions, but will also alter soil properties (including local soil microbiome) and modify ecological interactions that are crucial for the existence of plants. Moreover, climate change can directly disrupt or destroy mutualistic ecological interactions between species even before climate-driven extinction^75^.

Here the epiphytic orchid, *D. lindenii*, was used as an example of a plant the occurrence and survival of which is strictly related to the well-being of its host trees. The nature of the soil does not directly affect the ghost orchid, but can affect the distribution of its phorophytes and this topic should be further studied to more precisely evaluate the future chances of the survival of the host trees of *D. lindenii*. Epiphytic orchids depend on three main ecological partners: (1) phorophyte, (2) pollinator(s) and (3) microbiome and fungal associations.

Currently, the relationship between epiphytic orchids and their host tree(s) appear to be of little interest, despite the importance of this association for biodiversity conservation. It is noteworthy that about 60% of all vascular epiphytic plants are orchids^76,77^ and phorophyte trees constitute essential elements of numerous ecosystems by providing suitable substrates and environmental conditions for a large variety of plants to grow^77^. Because orchid identification is usually restricted to flowering individuals, little is known about the mechanisms that enable orchids to establish themselves in a specific zone of the tree canopy^78^. Wind-dispersed orchid seeds land randomly in the crowns of trees and their germination and further growth and development in a specific tree zone^79^ depends on micro-conditions of the host, e.g. microclimate, bark structure, pH, moss and lichen coverage and microbiome^80^. Currently phorophyte specificity of *D. lindenii* is limited to the identification of the tree on which the orchid grows^45^ and the microhabitat preferences of the orchid are unknown.

It is noteworthy that the ghost orchid occupies different phorophytes depending on local climatic conditions. In south Florida, where occasional sub-zero temperatures in winter are reported, *D. lindenii* occurs in areas where the relative humidity is high and vegetation dense^43^. On the other hand, in Western Cuba where sub-zero temperatures do not occur, *D. lindenii* grows on the bark of mixed semi-deciduous trees where there is little or no standing water^45^. Therefore, as it is expected that global warming will affect evaporation and modify the water cycle, *D. lindenii* will be affected in both parts of its geographical range.

Most orchids are cross-pollinated and as recently summarized, epiphytic orchids are characterized by more specialist pollinators than terrestrial orchids and self-pollination is less common in epiphytic species^38^. For this reason models of the future distribution of the climatic niches of orchids should be combined with simulations of changes in the availability of their pollinators^81^. As indicated previously, spatial shifts in the ranges of orchids and their pollinators are expected to occur^81,82^. Moreover, recently the desynchronization in the activity of pollen vectors and plant flowering time caused by global warming has become a topic of particular interest to ecologists^83–86^. Mismatches in phenology can be harmful for both the plants and pollinators, by causing a reduction in the incidence of pollination for the plant or starvation of the pollinator^75^. Based on reports in iNaturalist *D. lindenii* begins flowering in May/June and is still found in flower in August. Adults of *Dolba hyloeus* are recorded between March and October. Most adult *Cocytius antaeus* are reported between January and March, but it is not uncommon to see them in other months. Adult *Pachylia ficus* are active throughout the year according to the data available in iNaturalist. Due to the long activity period of all three pollinators of *D. lindenii*, the desynchronization and reduction in the incidence of pollination of this orchid as a result of global warming is unlikely to be important (Supplementary Annex 11). However, in the Mediterranean region, the development of the larvae of moths is likely to be sensitive to variation in temperature as they cannot avoid high temperatures and drought by becoming dormant^87^. Increasing incidence of high temperatures and extreme droughts due to global warming are likely to result in a decline in their abundance^87^, which is likely to have negative effects on plants pollinated by moths, like *D. lindenii*.

There is another potentially harmful effect of higher global temperatures. As recently shown^88^, they can change the characteristics of nectar, such as volume and sugar concentration to which nectar feeders are adapted. Nectar of *D. lindenii* contains three sugars (glucose, fructose and sucrose), three acids (lactic, malic and threonic) and 4-hydroxyl benzyl alcohol^89^. As summarized by Willmer^90^, nectar with a sugar concentration above 30–40% is difficult to imbibe for most Lepidoptera because of their long tongues and they require the nectar to be dilute and non-viscous. Currently the effects of higher temperatures on nectar production and composition are unknown, but in view of global warming this topic deserves further attention.

Moreover, little is known about the endophytes and mycorrhizal fungi of *D. lindenii.* The germination mycobiont of ghost orchid is a species of *Ceratobasidium,* but the species has yet to be identified^91^. As the species of the fungal partner of the ghost orchid is unknown it could not be used in the niche modelling. Without a doubt, *D. lindenii* depends on mycorrhizal fungi and other endophytes for nutrients^92–94^. Roots of the ghost orchid are usually colonized by mycorrhizal fungal pelotons^45^, but the composition of the microbes inhabiting the internal tissues of *D. lindenii* (other than roots) is unknown. This important feature of ghost orchid biology also needs to be studied and incorporated into niche modelling.

As evaluated in this study, *D. lindenii* and most of its pollinators and host trees are characterized by a narrow temperature (both annual and seasonality), but broad rainfall tolerance. The predicted increase in temperature in the area studied due to climate change can therefore significantly affect the distribution of this species, which is likely to result in local extinctions and range shifts. Analyses presented in this study indicate that the range of *D. lindenii* is likely to be significantly reduced, but within its reduced geographical range both its phorophytes and pollinators will occur so it is likely to survive the global warming. The future areas suitable for the coexistence of this orchid and its mutualistic partners should be thought of as potential refugia for the endangered ghost orchid.

## Conclusions

Species distribution models are useful tools for identifying climatic refugia of rare and endangered species. However, climate-driven range shifts and local extinctions can result in spatial mismatch with symbiotic organisms and reduce or impose constraints on species fitness. For this reason the modelling of the future distribution of any species should be combined with an evaluation of the availability of its ecological partners. For epiphytic orchids with specialist pollinators and a limited number of preferred phorophytes, both pollen vectors and host tree species must be incorporated in the modelling of potential refugial areas. Other ecological factors that should be incorporated are plant fungal partners that are crucial for orchid seed germination and supplying nutrients to ghost orchid plants. There is a need for further research on orchid endophytes and the isolation of fungal strains from orchids to be complemented by species-level identification of the mycobionts. A better understanding of spatial distribution of plant symbiotic fungi is crucial for evaluating their occurrence under global warming and for identifying potential refugia for rare plants.

### Supplementary Information

Supplementary Information 1.

Supplementary Information 2.

Supplementary Information 3.

Supplementary Information 4.

Supplementary Information 5.

Supplementary Information 6.

Supplementary Information 7.

Supplementary Information 8.

Supplementary Information 9.

Supplementary Information 10.

Supplementary Information 11.

## Data Availability

All relevant data are presented in the manuscript and supplementary files.
