# Supplementary material for: Future distribution of the epiphytic leafless orchid (Dendrophylax lindenii), its pollinators and phorophytes evaluated using niche modelling and three different climate change projections
Source: Sci Rep. 2023 Sep 14;13:15242. doi: 10.1038/s41598-023-42573-5 (PMC10502118; doi:10.1038/s41598-023-42573-5)
Supplement: Supplementary file 1 — Supplementary Information 1. [file 41598_2023_42573_MOESM1_ESM.pdf]

Future of epiphytic, leafless orchid (*Dendrophylax lindenii*) – complex modelling of the orchid, its pollinators and phorophytes

Marta Kolanowska[a*]

[a] University of Lodz, Faculty of Biology and Environmental Protection, Department of Geobotany and Plant Ecology, Banacha 12/16, 90-237 Lodz, Poland

* Corresponding author

Supplementary Annex 1. Locations of species studied used in ENM analyses. Maps created in ArcGIS.

**Species**	**Longitude**	**Latitude**
Annona glabra	-61.583728	16.274275
Annona glabra	-80.415169	27.756317
Annona glabra	-87.06607	20.633225
Annona glabra	-64.716448	18.345356
Annona glabra	-80.464517	27.808455
Annona glabra	-80.161544	27.190003
Annona glabra	-80.533997	28.009817
Annona glabra	-67.183754	18.16504
Annona glabra	-82.558555	27.301547
Annona glabra	-61.768631	16.068197
Annona glabra	-70.370823	19.709576
Annona glabra	-80.04853	26.67712
Annona glabra	-60.911442	14.464677
Annona glabra	-73.73239	18.199389
Annona glabra	-61.706964	15.949107
Annona glabra	-66.013948	18.441726
Annona glabra	-90.408333	20.875
Annona glabra	-86.966667	20.5
Annona glabra	-91.258333	20.675
Annona glabra	-96.367	19.583
Annona glabra	-96.1125	19.099722
Annona glabra	-91.182833	18.979139
Annona glabra	-91.890833	18.579083
Annona glabra	-105.017	19.383
Annona glabra	-74.326032	18.288323
Annona glabra	-65.882391	17.99734
Annona glabra	-68.9	18.4
Annona glabra	-73.888722	7.218111
Annona glabra	-84.105386	9.933671
Annona glabra	-92.155556	14.973611
Annona glabra	-84.163284	9.969964
Annona glabra	-76.717612	8.082643
Annona glabra	-74.994889	9.055206
Annona glabra	-83.21667	8.68333
Annona glabra	-75.363045	9.248975
Annona glabra	-87.062773	12.820023
Annona glabra	-68.875457	12.097945
Annona glabra	-69.040021	12.252868
Annona glabra	-92.5767	14.87508
Annona glabra	-70.7	18.75
Annona glabra	-70.738028	6.893794
Annona glabra	-88.010203	13.170978
Annona glabra	-90.225	20.658333
Annona glabra	-87.948043	13.191933
Annona glabra	-77.15709	7.84267

Annona glabra	-99.733333	17.708333
Annona glabra	-70.218456	11.658468
Annona glabra	-97.028333	19.531667
Annona glabra	-90.29722	20.52222
Annona glabra	-87.022566	15.774984
Annona glabra	-79.862957	9.143723
Annona glabra	-90.36778	20.53889
Annona glabra	-85.11667	10.96667
Annona glabra	-99.760556	17.865278
Annona glabra	-90.420833	20.5375
Annona glabra	-77.198363	8.409167
Annona glabra	-77.319014	18.151419
Annona glabra	-100.5	17.085
Annona glabra	-96.16681	15.723246
Annona glabra	-98.854679	16.538639
Annona glabra	-90.333333	20.201389
Annona glabra	-90.408333	20.241667
Annona glabra	-101.641667	17.731944
Annona glabra	-82.816121	9.749106
Annona glabra	-69.12087	12.331863
Annona glabra	-99.840278	16.866667
Annona glabra	-83.506923	10.448877
Annona glabra	-100.204722	16.985278
Annona glabra	-96.626758	15.694342
Annona glabra	-91.468333	18.441944
Annona glabra	-100.148555	16.961111
Annona glabra	-82.344364	28.00568
Annona glabra	-90.292	18.633
Annona glabra	-90.37778	19.93583
Annona glabra	-82.564127	28.104046
Annona glabra	-91.5	18.25
Annona glabra	-91.75	18.366389
Annona glabra	-81.353813	27.65665
Annona glabra	-96.383	19.533
Annona glabra	-96.423611	19.605833
Annona glabra	-90.4775	19.879167
Annona glabra	-91.25575	18.957361
Annona glabra	-91.75	18.183
Annona glabra	-88.211501	21.568285
Annona glabra	-67.056379	18.044014
Annona glabra	-96.735555	19.536389
Annona glabra	-82.674175	27.500139
Annona glabra	-94.9469	16.56083
Annona glabra	-99.833333	16.8
Annona glabra	-91.52225	18.763806
Annona glabra	-88.75861	19.27889
Annona glabra	-82.500731	27.27759
Annona glabra	-91.8	18.417

Annona glabra	-67.158883	18.414517
Annona glabra	-89.34722	21.29944
Annona glabra	-89.875	20.508333
Annona glabra	-94.143333	18.143333
Annona glabra	-70.006815	18.58266
Annona glabra	-88.525	18.425
Annona glabra	-90.591667	19.325
Annona glabra	-94.1	18.05
Annona glabra	-88.308333	18.508333
Annona glabra	-82.422347	27.115603
Annona glabra	-103.69835	18.652403
Annona glabra	-88.475	18.491667
Annona glabra	-96.183	19.133
Annona glabra	-61.053705	14.758586
Annona glabra	-88.891667	21.125
Annona glabra	-88.441011	16.559272
Annona glabra	-88.583	20.717
Annona glabra	-88.791667	20.675
Annona glabra	-94.1	18.000833
Annona glabra	-101.332113	17.486355
Annona glabra	-88.958333	21.041667
Annona glabra	-66.85	18.48333
Annona glabra	-88.430198	16.605669
Annona glabra	-88.858333	20.908333
Annona glabra	-88.841667	18.091667
Annona glabra	-89.458333	20.841667
Annona glabra	-92.277222	18.644444
Annona glabra	-81.341435	27.382815
Annona glabra	-94.109722	17.955
Annona glabra	-94.1	17.9
Annona glabra	-88.391667	20.741667
Annona glabra	-88.608333	19.425
Annona glabra	-88.969021	17.408281
Annona glabra	-66.637828	18.481128
Annona glabra	-88.876944	17.938889
Annona glabra	-88.841667	17.991667
Annona glabra	-71.181723	17.960115
Annona glabra	-92.025	18.616667
Annona glabra	-80.608824	28.392622
Annona glabra	-81.434	26.770402
Annona glabra	-88.782778	21.017778
Annona glabra	-66.422824	18.469311
Annona glabra	-66.062825	18.401903
Annona glabra	-92.8	18.166667
Annona glabra	-92.64461	18.27736
Annona glabra	-105.233	21.15
Annona glabra	-66.33863	18.479968
Annona glabra	-95.067	18.567

Annona glabra	-92.55592	18.53508
Annona glabra	-66.134767	18.436692
Annona glabra	-65.999231	18.235298
Annona glabra	-98.197222	20.983889
Annona glabra	-94.626389	18.26
Annona glabra	-94.483611	17.939444
Annona glabra	-81.661308	26.708975
Annona glabra	-87.583333	20.081944
Annona glabra	-95.878611	18.746944
Annona glabra	-87.766667	21.3
Annona glabra	-81.594788	26.715428
Annona glabra	-95.017	18.533
Annona glabra	-92.2267	18.55528
Annona glabra	-87.808333	19.808333
Annona glabra	-88.041667	19.575
Annona glabra	-92.647774	18.405659
Annona glabra	-92.57687	18.44745
Annona glabra	-61.5036	16.221866
Annona glabra	-80.775676	27.726738
Annona glabra	-81.904075	26.730287
Annona glabra	-92.646389	18.533611
Annona glabra	-87.993611	19.361389
Annona glabra	-92.84834	18.24742
Annona glabra	-105.221881	21.095341
Annona glabra	-87.994167	19.493056
Annona glabra	-94.596111	18.186389
Annona glabra	-66.049308	18.260587
Annona glabra	-92.718611	18.518056
Annona glabra	-80.587242	28.047928
Annona glabra	-80.640816	27.659841
Annona glabra	-65.800903	18.0937
Annona glabra	-61.526412	16.291486
Annona glabra	-81.763921	26.693724
Annona glabra	-87.708333	20.508333
Annona glabra	-105.25	20.78333
Annona glabra	-61.016119	14.684001
Annona glabra	-81.81092	26.711113
Annona glabra	-80.611339	28.181835
Annona glabra	-97.403194	21.581111
Annona glabra	-61.165804	14.701391
Annona glabra	-61.407985	16.217935
Annona glabra	-80.541	27.150631
Annona glabra	-81.612373	26.605995
Annona glabra	-61.62836	15.9709
Annona glabra	-96.436667	19.801667
Annona glabra	-65.90819	18.420923
Annona glabra	-86.9991317	20.320107
Annona glabra	-82.113742	26.635279

Annona glabra	-87.140821	20.597455
Annona glabra	-86.908333	20.508333
Annona glabra	-61.56885	16.12372
Annona glabra	-61.220351	14.793664
Annona glabra	-75.869255	21.112509
Annona glabra	-81.349252	26.612929
Annona glabra	-65.798522	18.395978
Annona glabra	-86.845278	20.445556
Annona glabra	-61.25	15.33333
Annona glabra	-61.769467	16.148848
Annona glabra	-65.697729	18.360771
Annona glabra	-61.633811	15.857321
Annona glabra	-105.206608	21.529325
Annona glabra	-65.280328	18.312953
Annona glabra	-86.965588	20.758022
Annona glabra	-81.561354	26.457962
Annona glabra	-81.823167	26.57353
Annona glabra	-80.501878	27.948447
Annona glabra	-81.89921	26.659894
Annona glabra	-61.724602	16.333327
Annona glabra	-75.972053	23.662113
Annona glabra	-60.920167	13.7753
Annona glabra	-87.094444	21.208333
Annona glabra	-80.375847	27.649837
Annona glabra	-61.823963	17.646442
Annona glabra	-61.094527	14.612046
Annona glabra	-81.484184	26.432268
Annona glabra	-105.298889	20.726389
Annona glabra	-80.38333	27.7
Annona glabra	-80.474975	27.902803
Annona glabra	-82.112899	26.445558
Annona glabra	-81.913825	26.608367
Annona glabra	-81.608933	26.372545
Annona glabra	-82.04608	26.446292
Annona glabra	-61.772334	16.348764
Annona glabra	-81.882207	26.525174
Annona glabra	-60.850679	14.437593
Annona glabra	-80.34668	27.352495
Annona glabra	-81.981445	26.543154
Annona glabra	-81.96744	26.476308
Annona glabra	-81.842203	26.444932
Annona glabra	-81.774364	26.463211
Annona glabra	-81.631788	26.315822
Annona glabra	-80.911811	26.750499
Annona glabra	-105.908116	22.780749
Annona glabra	-81.1465	26.268592
Annona glabra	-80.314025	27.228138
Annona glabra	-80.998833	26.323775

Annona glabra	-81.822656	26.381545
Annona glabra	-81.611543	26.09508
Annona glabra	-80.248906	27.102411
Annona glabra	-81.815198	26.29887
Annona glabra	-81.719752	26.202488
Annona glabra	-81.391096	25.984282
Annona glabra	-80.250867	27.154218
Annona glabra	-81.265787	26.096308
Annona glabra	-80.18833	27.141131
Annona glabra	-81.594554	25.988879
Annona glabra	-81.510502	25.959951
Annona glabra	-81.196636	26.1661
Annona glabra	-80.231157	27.222584
Annona glabra	-80.896287	26.17675
Annona glabra	-81.705859	26.110756
Annona glabra	-81.784867	26.244609
Annona glabra	-81.298576	25.926437
Annona glabra	-80.371399	25.43476
Annona glabra	-76.212454	25.187129
Annona glabra	-80.5333	25.6167
Annona glabra	-80.185643	26.947208
Annona glabra	-81.260381	25.989398
Annona glabra	-81.771619	26.107655
Annona glabra	-80.94072	25.92683
Annona glabra	-80.135636	27.007161
Annona glabra	-80.307104	26.85891
Annona glabra	-80.714408	26.148161
Annona glabra	-81.150178	25.867705
Annona glabra	-80.309696	25.622775
Annona glabra	-81.100656	25.861047
Annona glabra	-80.215275	26.852395
Annona glabra	-80.632242	26.147214
Annona glabra	-81.78375	26.174495
Annona glabra	-80.45555	25.666283
Annona glabra	-80.677544	25.3939
Annona glabra	-80.950729	25.848913
Annona glabra	-81.033067	25.857062
Annona glabra	-81.099786	25.788433
Annona glabra	-80.541019	25.667711
Annona glabra	-80.819703	25.687845
Annona glabra	-80.766741	25.709127
Annona glabra	-80.577392	25.757837
Annona glabra	-80.727768	25.39377
Annona glabra	-80.849571	25.826395
Annona glabra	-80.606161	25.383011
Annona glabra	-80.232312	26.632901
Annona glabra	-80.166962	26.84197
Annona glabra	-77.363905	25.021509

Annona glabra	-81.030686	25.760158
Annona glabra	-80.964387	25.749884
Annona glabra	-80.482105	25.374655
Annona glabra	-80.766097	25.657653
Annona glabra	-80.910453	25.761205
Annona glabra	-80.452085	25.882234
Annona glabra	-80.448871	26.061527
Annona glabra	-80.697937	25.543977
Annona glabra	-80.365551	26.221745
Annona glabra	-80.211303	26.501122
Annona glabra	-80.116581	26.934828
Annona glabra	-80.484542	25.762283
Annona glabra	-80.119614	26.8863
Annona glabra	-80.830643	25.764843
Annona glabra	-77.398482	25.067133
Annona glabra	-80.27768	25.681812
Annona glabra	-80.434287	26.014631
Annona glabra	-80.7629	25.754095
Annona glabra	-80.144822	26.786683
Annona glabra	-80.783762	25.441595
Annona glabra	-80.061936	26.861222
Annona glabra	-80.41433	26.137858
Annona glabra	-80.224394	26.380143
Annona glabra	-80.392302	25.777108
Annona glabra	-80.169477	26.722864
Annona glabra	-80.151157	25.716137
Annona glabra	-80.166705	26.655826
Annona glabra	-80.362938	26.128695
Annona glabra	-80.240137	26.282062
Annona glabra	-80.369212	25.829924
Annona glabra	-80.247588	26.052283
Annona glabra	-80.832038	25.323576
Annona glabra	-80.088176	26.803713
Annona glabra	-80.316108	26.06642
Annona glabra	-62.204802	16.802648
Annona glabra	-80.102996	26.152375
Annona glabra	-80.16188	26.486292
Annona glabra	-80.309079	26.14205
Annona glabra	-80.38375	26.014386
Annona glabra	-80.195888	26.138105
Annona glabra	-80.338758	25.950056
Annona glabra	-80.090834	26.260945
Annona glabra	-80.081949	26.607889
Annona glabra	-80.191003	26.29343
Annona glabra	-80.186772	26.229198
Annona glabra	-80.115993	26.387495
Annona glabra	-80.236661	25.902285
Annona glabra	-80.302087	26.012419

Annona glabra	-80.16079	25.902212
Annona glabra	-80.093194	26.549549
Annona glabra	-80.158067	26.57383
Annona glabra	-80.201189	25.853728
Annona glabra	-80.176872	26.088791
Annona glabra	-80.30559	25.819314
Annona glabra	-80.308747	25.77391
Annona glabra	-80.239141	25.783457
Annona glabra	-80.155511	25.669158
Annona glabra	-81.379272	24.703363
Cocytius antaeus	-104.246655	23.3867
Cocytius antaeus	-99.05789	19.484304
Cocytius antaeus	-83.969641	9.940678
Cocytius antaeus	-99.216667	19.216667
Cocytius antaeus	-99.133056	19.433056
Cocytius antaeus	-99.166667	19.283333
Cocytius antaeus	-101.307652	20.703495
Cocytius antaeus	-98.438098	18.912924
Cocytius antaeus	-74.949671	10.988628
Cocytius antaeus	-75.059534	9.072266
Cocytius antaeus	-101.359683	20.682369
Cocytius antaeus	-92.225556	16.467222
Cocytius antaeus	-98.945	18.823333
Cocytius antaeus	-92.607778	16.707222
Cocytius antaeus	-99.229462	23.774978
Cocytius antaeus	-97.783333	19.866667
Cocytius antaeus	-97.225556	16.838611
Cocytius antaeus	-98.983333	18.766667
Cocytius antaeus	-92.134167	16.251111
Cocytius antaeus	-99.10531	18.991308
Cocytius antaeus	-92.3837895919	16.4009010666
Cocytius antaeus	-89.35357	14.56689
Cocytius antaeus	-99.19	18.943333
Cocytius antaeus	-87.074979	14.035069
Cocytius antaeus	-79.472806	9.059332
Cocytius antaeus	-103.730833	19.243611
Cocytius antaeus	-97.920278	20.264167
Cocytius antaeus	-97.100833	18.815
Cocytius antaeus	-99.503333	17.555
Cocytius antaeus	-103.725	19.315556
Cocytius antaeus	-66.576727	18.237192
Cocytius antaeus	-91.568333	16.900833
Cocytius antaeus	-97.6075	19.919722
Cocytius antaeus	-94.85	18.266667
Cocytius antaeus	-96.997778	18.896944
Cocytius antaeus	-96.973934	19.50256
Cocytius antaeus	-99.447778	17.345833

Cocytius antaeus	-96.982	17.898
Cocytius antaeus	-95.056389	18.472222
Cocytius antaeus	-93.033333	16.733333
Cocytius antaeus	-95.348333	18.455
Cocytius antaeus	-96.941667	19.421389
Cocytius antaeus	-99.145556	23.031944
Cocytius antaeus	-89.643538	21.024236
Cocytius antaeus	-88.974967	17.097365
Cocytius antaeus	-76.748358	18.027881
Cocytius antaeus	-73.134658	18.465755
Cocytius antaeus	-67.127817	18.082245
Cocytius antaeus	-105.081667	19.53
Cocytius antaeus	-89.499579	21.079384
Cocytius antaeus	-96.38781	18.83775
Cocytius antaeus	-75.811347	20.025672
Cocytius antaeus	-97.871667	20.389722
Cocytius antaeus	-61.523034	16.228983
Cocytius antaeus	-81.368758	19.299178
Cocytius antaeus	-76.906953	18.385606
Cocytius antaeus	-61.06627	14.51991
Cocytius antaeus	-95.066667	18.583333
Cocytius antaeus	-95.099722	18.419167
Cocytius antaeus	-81.168094	19.315861
Cocytius antaeus	-87.111034	20.584745
Cocytius antaeus	-65.754443	18.387828
Cocytius antaeus	-81.397856	26.114812
Cocytius antaeus	-106.442357	23.300682
Comocladia dentata	-69.58333	18.41666
Comocladia dentata	-71.03	18.8083
Comocladia dentata	-72.4903	18.9756
Comocladia dentata	-71.232783	18.117481
Comocladia dentata	-71.67	18.325
Comocladia dentata	-71.638702	17.903768
Comocladia dentata	-66.7	18.33333
Comocladia dentata	-72.104415	18.833194
Comocladia dentata	-66.3242	18.2761
Comocladia dentata	-71.56666	17.93333
Comocladia dentata	-69.26666	18.46666
Comocladia dentata	-66.98333	18.31666
Comocladia dentata	-69.9333	18.48333
Comocladia dentata	-69.77333	18.46972
Comocladia dentata	-69.66861	18.45083
Comocladia dentata	-69.226186	18.991752
Comocladia dentata	-80.646135	22.24755
Comocladia dentata	-81.10933	19.315692
Comocladia dentata	-81.191338	19.313708
Comocladia dentata	-82.287619	22.718349
Comocladia dentata	-84.133525	22.116517

Comocladia dentata	-77.897433	21.555223
Comocladia dentata	-82.639578	22.949596
Comocladia dentata	-81.861255	22.909878
Comocladia dentata	-83.418899	22.516062
Comocladia dentata	-82.326817	23.155925
Dendrophylax lindenii	-81.6942228397	25.804938848
Dendrophylax lindenii	-84.8800722664	21.9527930862
Dendrophylax lindenii	-78.1851290801	21.7042660661
Dendrophylax lindenii	-81.9535	26.663
Dendrophylax lindenii	-81.6810888461	26.3954440796
Dendrophylax lindenii	-81.614342	26.380144
Dendrophylax lindenii	-81.6222630139	26.3019804776
Dendrophylax lindenii	-81.759716798	26.369976512
Dendrophylax lindenii	-81.6153781257	26.2014961513
Dendrophylax lindenii	-81.7141662462	26.3122996235
Dendrophylax lindenii	-81.1455099248	26.2846470133
Dendrophylax lindenii	-81.2881123308	26.1676238503
Dendrophylax lindenii	-81.776846528	26.2996319875
Dendrophylax lindenii	-81.5047652578	26.0462386598
Dendrophylax lindenii	-81.6630989796	26.2558780378
Dendrophylax lindenii	-81.5669837966	26.1143579222
Dendrophylax lindenii	-81.7636031104	26.2493172812
Dendrophylax lindenii	-81.407542	26.041375
Dendrophylax lindenii	-81.2456098452	26.0252821031
Dendrophylax lindenii	-81.0018506199	25.9254941159
Dendrophylax lindenii	-81.7969118871	26.1972983436
Dendrophylax lindenii	-81.1299471964	25.9030070744
Dendrophylax lindenii	-80.8893330959	26.0522032606
Diospyros crassinervis	-69.628026	19.105702
Diospyros crassinervis	-70.733333	19.816667
Diospyros crassinervis	-74.85	20.016667

Diospyros crassinervis	-77.25	21.533333
Diospyros crassinervis	-79.983333	21.75
Diospyros crassinervis	-74.333333	22.616667
Diospyros crassinervis	-82.533333	23.083333
Diospyros crassinervis	-82.466667	23.1
Diospyros crassinervis	-75.916667	23.45
Diospyros crassinervis	-75.866667	23.466667
Diospyros crassinervis	-74.566667	23.95
Diospyros crassinervis	-77.55	24.116667
Diospyros crassinervis	-75.35	24.25
Diospyros crassinervis	-75.5492	24.4706
Diospyros crassinervis	-77.883333	24.833333
Diospyros crassinervis	-77.9244	24.8922
Diospyros crassinervis	-76.233333	25.166667
Diospyros crassinervis	-71.56833	18.28555
Diospyros crassinervis	-72.166667	18.683333
Diospyros crassinervis	-80.466667	22.05
Diospyros crassinervis	-76.916667	20.116667
Diospyros crassinervis	-72.666667	19.666667
Diospyros crassinervis	-80.95277	22.05417
Diospyros crassinervis	-70.25	19.633333
Diospyros crassinervis	-83.116667	22.75
Diospyros crassinervis	-75.716667	22.183333
Diospyros crassinervis	-69.316667	19.25
Diospyros crassinervis	-80.89444	22.04167
Diospyros crassinervis	-70.983333	19.35
Diospyros crassinervis	-69.433333	19.083333
Diospyros crassinervis	-71.55	19.483333
Diospyros crassinervis	-71.166667	19.416667
Diospyros crassinervis	-76.42889	21.28028
Diospyros crassinervis	-76.283333	20.883333
Diospyros crassinervis	-73.983333	22.416667
Diospyros crassinervis	-74.1736	22.7161
Diospyros crassinervis	-83.166667	21.583333
Diospyros crassinervis	-75.916667	20.383333
Diospyros crassinervis	-82.833333	21.666667
Diospyros crassinervis	-82.716667	21.683333
Diospyros crassinervis	-74.25	22.766667
Diospyros crassinervis	-74.35	22.833333
Diospyros crassinervis	-77.65	21.733333
Diospyros crassinervis	-82.55	21.566667
Diospyros crassinervis	-83.333333	22.666667
Diospyros crassinervis	-75.733333	20.833333
Diospyros crassinervis	-77.533333	23.95
Diospyros crassinervis	-75.916667	23.583333
Diospyros crassinervis	-77.55	24.016667
Diospyros crassinervis	-75.1597	23.4069
Diospyros crassinervis	-79.833333	22.333333

Diospyros crassinervis	-83.44028	22.78222
Diospyros crassinervis	-75.966667	23.45
Diospyros crassinervis	-75.85	23.516667
Diospyros crassinervis	-75.666667	20.516667
Diospyros crassinervis	-75.083333	23.166667
Diospyros crassinervis	-75.416667	24.333333
Diospyros crassinervis	-75.3333	23.675
Diospyros crassinervis	-76.366667	24.05
Diospyros crassinervis	-75.5164	24.4044
Diospyros crassinervis	-83.066667	22.633333
Diospyros crassinervis	-74.483333	23.966667
Diospyros crassinervis	-82.016667	23.05
Diospyros crassinervis	-81.683333	23.083333
Diospyros crassinervis	-76.15	24.85
Diospyros crassinervis	-74.516667	24.033333
Diospyros crassinervis	-82.716667	23.016667
Diospyros crassinervis	-74.502387	24.108087
Diospyros crassinervis	-77.7404	24.512
Diospyros crassinervis	-82.033333	23.1
Diospyros crassinervis	-83.716667	22.766667
Diospyros crassinervis	-83.316667	22.916667
Diospyros crassinervis	-82.283333	23.116667
Diospyros crassinervis	-82.25	23.166667
Diospyros crassinervis	-77.766667	24.683333
Diospyros crassinervis	-80.268333	25.675
Diospyros crassinervis	-78.016667	25.133333
Dolba hyloeus	-97.305028	30.837448
Dolba hyloeus	-85.874034	30.811253
Dolba hyloeus	-93.795837	30.828178
Dolba hyloeus	-80.1796607	27.1803315
Dolba hyloeus	-97.81243	30.65105
Dolba hyloeus	-97.695917	30.626105
Dolba hyloeus	-98.023819	30.50785
Dolba hyloeus	-98.613962	29.880672
Dolba hyloeus	-97.964368	30.511823
Dolba hyloeus	-98.08012	30.203994
Dolba hyloeus	-97.76274	30.508255
Dolba hyloeus	-97.999222	30.230798
Dolba hyloeus	-98.544371	29.812812
Dolba hyloeus	-97.841731	30.427764
Dolba hyloeus	-97.787812	30.148383
Dolba hyloeus	-97.886526	30.208921
Dolba hyloeus	-97.025803	30.383749
Dolba hyloeus	-96.689925	30.54173
Dolba hyloeus	-97.293029	30.070296
Dolba hyloeus	-97.722824	30.187186
Dolba hyloeus	-97.202777	30.449568
Dolba hyloeus	-98.088554	29.705123

Dolba hyloeus	-97.756988	30.418972
Dolba hyloeus	-97.784705	30.373775
Dolba hyloeus	-97.774278	30.196884
Dolba hyloeus	-97.93939	29.888679
Dolba hyloeus	-97.82695	30.244819
Dolba hyloeus	-91.21136	30.791061
Dolba hyloeus	-97.737137	30.356183
Dolba hyloeus	-97.182635	30.164488
Dolba hyloeus	-98.161835	29.71174
Dolba hyloeus	-96.100284	30.388311
Dolba hyloeus	-97.781325	30.284055
Dolba hyloeus	-97.670595	30.299688
Dolba hyloeus	-96.214105	30.562411
Dolba hyloeus	-96.337128	30.556997
Dolba hyloeus	-97.296745	30.134422
Dolba hyloeus	-96.276962	30.599342
Dolba hyloeus	-97.733968	30.285858
Dolba hyloeus	-98.703998	29.509991
Dolba hyloeus	-97.575613	29.620006
Dolba hyloeus	-90.93607	30.539202
Dolba hyloeus	-98.253446	29.572551
Dolba hyloeus	-98.724022	29.379255
Dolba hyloeus	-83.869867	30.535072
Dolba hyloeus	-96.269942	30.175626
Dolba hyloeus	-98.619897	29.58428
Dolba hyloeus	-91.092594	30.535652
Dolba hyloeus	-98.514073	29.585861
Dolba hyloeus	-95.606066	30.697428
Dolba hyloeus	-95.728245	30.637551
Dolba hyloeus	-95.445248	30.808721
Dolba hyloeus	-88.944728	30.601541
Dolba hyloeus	-96.498178	29.97733
Dolba hyloeus	-98.446129	29.475637
Dolba hyloeus	-91.104556	30.369999
Dolba hyloeus	-94.222422	30.720953
Dolba hyloeus	-98.570717	29.492247
Dolba hyloeus	-95.776467	30.366117
Dolba hyloeus	-94.951851	30.701954
Dolba hyloeus	-91.1803	30.41487
Dolba hyloeus	-88.223647	30.664288
Dolba hyloeus	-91.096158	30.310698
Dolba hyloeus	-93.898203	30.662522
Dolba hyloeus	-82.553795	30.318262
Dolba hyloeus	-94.378278	30.580997
Dolba hyloeus	-95.356949	30.402253
Dolba hyloeus	-91.929163	30.101759
Dolba hyloeus	-88.70914	30.434242
Dolba hyloeus	-95.662885	30.287036

Dolba hyloeus	-95.74073	30.067892
Dolba hyloeus	-96.111013	29.81713
Dolba hyloeus	-95.763091	30.166506
Dolba hyloeus	-94.837864	30.486795
Dolba hyloeus	-88.653541	30.451667
Dolba hyloeus	-95.682063	30.022595
Dolba hyloeus	-95.500232	30.325617
Dolba hyloeus	-95.585687	30.19158
Dolba hyloeus	-94.386788	30.457964
Dolba hyloeus	-85.009635	29.93826
Dolba hyloeus	-88.41105	30.439635
Dolba hyloeus	-95.605437	30.01708
Dolba hyloeus	-95.479888	30.209612
Dolba hyloeus	-94.19072	30.337575
Dolba hyloeus	-95.745279	29.718504
Dolba hyloeus	-95.448517	30.122989
Dolba hyloeus	-86.950005	30.428536
Dolba hyloeus	-88.138399	30.381006
Dolba hyloeus	-95.386458	30.128595
Dolba hyloeus	-82.37065	29.636258
Dolba hyloeus	-87.635422	30.34757
Dolba hyloeus	-95.558372	29.998297
Dolba hyloeus	-94.765931	30.098168
Dolba hyloeus	-82.22017	29.733278
Dolba hyloeus	-95.421849	30.053706
Dolba hyloeus	-82.275062	29.654005
Dolba hyloeus	-95.65	29.682
Dolba hyloeus	-94.815908	30.004966
Dolba hyloeus	-95.589474	29.760448
Dolba hyloeus	-94.947159	29.768991
Dolba hyloeus	-95.49707	29.711018
Dolba hyloeus	-85.154236	29.654087
Dolba hyloeus	-95.44963	29.763924
Dolba hyloeus	-95.451847	29.699842
Dolba hyloeus	-95.335579	29.776484
Dolba hyloeus	-95.011978	29.577608
Dolba hyloeus	-81.70849	30.052809
Dolba hyloeus	-95.381213	29.750002
Dolba hyloeus	-81.638261	29.951847
Dolba hyloeus	-94.958231	29.424667
Dolba hyloeus	-95.419642	29.042732
Dolba hyloeus	-96.991371	28.145491
Dolba hyloeus	-96.534405	28.434145
Dolba hyloeus	-81.262542	29.643928
Dolba hyloeus	-90.662511	29.254315
Dolba hyloeus	-81.62509	28.355138
Dolba hyloeus	-81.479588	28.385199
Dolba hyloeus	-81.352539	27.178904

Dolba hyloeus	-81.375205	27.716288
Dolba hyloeus	-81.022986	27.565825
Dolba hyloeus	-80.120186	26.90517
Dolba hyloeus	-80.899456	25.286605
Erythroxylum areolatum	-66.962067	17.941595
Erythroxylum areolatum	-67.21225	17.969919
Erythroxylum areolatum	-86.883	20.833
Erythroxylum areolatum	-96.367	19.6
Erythroxylum areolatum	-96.456389	19.933333
Erythroxylum areolatum	-105.016667	19.416667
Erythroxylum areolatum	-75.8574	9.77823
Erythroxylum areolatum	-75.75	9.74
Erythroxylum areolatum	-89.83208	13.82617
Erythroxylum areolatum	-75.431243	10.353278
Erythroxylum areolatum	-75.789223	10.161358
Erythroxylum areolatum	-100.101944	18.951667
Erythroxylum areolatum	-104.5	22.266667
Erythroxylum areolatum	-92.270556	16.373333
Erythroxylum areolatum	-74.70537	10.99863
Erythroxylum areolatum	-100.283333	18.380278
Erythroxylum areolatum	-75.695609	10.136033
Erythroxylum areolatum	-75.644775	10.179153
Erythroxylum areolatum	-104.6225	19.477778
Erythroxylum areolatum	-90.375	20.508333
Erythroxylum areolatum	-95.92861	15.89028
Erythroxylum areolatum	-90.276453	20.284687
Erythroxylum areolatum	-96.169444	15.752778
Erythroxylum areolatum	-89.40029	14.36256
Erythroxylum areolatum	-96.23056	15.69722
Erythroxylum areolatum	-90.34167	20.19444
Erythroxylum areolatum	-66.904511	17.954284
Erythroxylum areolatum	-96.765	19.33583
Erythroxylum areolatum	-89.20056	19.2125
Erythroxylum areolatum	-96.414722	19.533889
Erythroxylum areolatum	-91.283333	16.783333
Erythroxylum areolatum	-66.8583	17.97487
Erythroxylum areolatum	-91.84167	17.74722
Erythroxylum areolatum	-95.016667	18.25
Erythroxylum areolatum	-89.06083	19.52083
Erythroxylum areolatum	-91.538333	17.8
Erythroxylum areolatum	-71.388502	17.686518
Erythroxylum areolatum	-92.016667	17.666667
Erythroxylum areolatum	-96.681667	18.9875
Erythroxylum areolatum	-95.189722	18.460556
Erythroxylum areolatum	-94.143333	18.143333
Erythroxylum areolatum	-89.84833	18.60583
Erythroxylum areolatum	-94.34972	18.10111
Erythroxylum areolatum	-96.383	19.367

Erythroxylum areolatum	-89.9065	18.54267
Erythroxylum areolatum	-90.83	16.648611
Erythroxylum areolatum	-92.043056	17.484722
Erythroxylum areolatum	-90.725	18.608333
Erythroxylum areolatum	-89.84833	18.30222
Erythroxylum areolatum	-80.337979	22.132364
Erythroxylum areolatum	-96.35	18.85
Erythroxylum areolatum	-71.152952	17.996278
Erythroxylum areolatum	-67.9033	18.05914
Erythroxylum areolatum	-89.55717	14.33356
Erythroxylum areolatum	-97.775833	22.118611
Erythroxylum areolatum	-96.417	19.75
Erythroxylum areolatum	-87.812083	19.722278
Erythroxylum areolatum	-87.475	20.191667
Erythroxylum areolatum	-95.066389	18.583611
Erythroxylum areolatum	-95.016667	18.516667
Erythroxylum areolatum	-96.291667	18.308333
Erythroxylum areolatum	-87.6	19.783333
Erythroxylum areolatum	-88.041944	19.585833
Erythroxylum areolatum	-81.167505	19.315028
Erythroxylum areolatum	-87.7347588	20.4957415
Erythroxylum areolatum	-97.382194	21.570278
Erythroxylum areolatum	-86.833611	21.161111
Erythroxylum areolatum	-105.3	20.78333
Erythroxylum areolatum	-87.058333	20.858333
Erythroxylum areolatum	-86.908333	20.975
Erythroxylum areolatum	-98.8946298	21.635624
Erythroxylum areolatum	-106.411667	23.286667
Erythroxylum areolatum	-80.27194	25.67694
Erythroxylum areolatum	-77.2667	26.6743
Fraxinus caroliniana	-81.935856	30.821253
Fraxinus caroliniana	-87.635136	30.866441
Fraxinus caroliniana	-89.7402	30.5395
Fraxinus caroliniana	-89.5552	30.7476
Fraxinus caroliniana	-89.59428	30.43938
Fraxinus caroliniana	-91.006653	30.599955
Fraxinus caroliniana	-88.775612	30.73403
Fraxinus caroliniana	-89.813	30.6785
Fraxinus caroliniana	-89.966187	30.507503
Fraxinus caroliniana	-89.706955	30.306913
Fraxinus caroliniana	-95.657718	30.253693
Fraxinus caroliniana	-82.945523	29.56551
Fraxinus caroliniana	-82.399202	29.888595
Fraxinus caroliniana	-84.876401	29.842666
Fraxinus caroliniana	-84.79332	29.834867
Fraxinus caroliniana	-82.367892	29.634655
Fraxinus caroliniana	-95.269427	30.052446
Fraxinus caroliniana	-83.052605	29.290963

Fraxinus caroliniana	-81.70958	30.296247
Fraxinus caroliniana	-81.758362	30.182563
Fraxinus caroliniana	-81.96575	29.253387
Fraxinus caroliniana	-81.486069	29.7241
Fraxinus caroliniana	-81.774269	29.475364
Fraxinus caroliniana	-90.504335	14.660683
Fraxinus caroliniana	-82.035106	28.316735
Fraxinus caroliniana	-82.239197	28.145981
Fraxinus caroliniana	-81.118758	28.680567
Fraxinus caroliniana	-82.14878	27.368462
Fraxinus caroliniana	-82.397542	27.246924
Fraxinus caroliniana	-81.170298	28.655425
Fraxinus caroliniana	-82.283097	27.267975
Fraxinus caroliniana	-82.336028	27.21594
Fraxinus caroliniana	-81.808652	27.624136
Fraxinus caroliniana	-82.377104	28.073644
Fraxinus caroliniana	-81.816441	27.902014
Fraxinus caroliniana	-81.053107	28.806705
Fraxinus caroliniana	-81.545646	27.47168
Fraxinus caroliniana	-80.996727	28.58028
Fraxinus caroliniana	-82.45053	27.402758
Fraxinus caroliniana	-82.4828	27.350677
Fraxinus caroliniana	-81.640488	28.560625
Fraxinus caroliniana	-82.539471	27.555739
Fraxinus caroliniana	-81.888885	27.226714
Fraxinus caroliniana	-81.42682	27.714008
Fraxinus caroliniana	-82.341157	27.127965
Fraxinus caroliniana	-82.502747	27.278542
Fraxinus caroliniana	-80.802459	28.533236
Fraxinus caroliniana	-81.435871	26.747623
Fraxinus caroliniana	-81.663808	26.708611
Fraxinus caroliniana	-81.699596	26.653919
Fraxinus caroliniana	-81.822303	26.574413
Fraxinus caroliniana	-81.545773	26.456064
Fraxinus caroliniana	-81.613815	26.371746
Fraxinus caroliniana	-80.347167	27.352312
Fraxinus caroliniana	-81.774433	26.461245
Fraxinus caroliniana	-81.62956	26.315951
Fraxinus caroliniana	-80.617346	26.988146
Fraxinus caroliniana	-81.591808	26.227223
Fraxinus caroliniana	-81.746528	26.336503
Fraxinus caroliniana	-81.503536	26.142265
Fraxinus caroliniana	-80.251022	27.107911
Fraxinus caroliniana	-81.409222	26.009662
Fraxinus caroliniana	-81.26527	26.095848
Fraxinus caroliniana	-81.4693	25.94675
Fraxinus caroliniana	-81.714281	26.077629
Fraxinus caroliniana	-80.169167	26.975

Fraxinus caroliniana	-81.774025	26.106817
Fraxinus caroliniana	-81.065946	25.858057
Fraxinus caroliniana	-81.149982	25.86735
Fraxinus caroliniana	-81.019591	25.854883
Fraxinus caroliniana	-80.224432	26.545021
Fraxinus caroliniana	-81.100484	25.788014
Fraxinus caroliniana	-80.108878	26.889595
Pachylia ficus	-82.125889	26.436688
Pachylia ficus	-82.681326	27.43381
Pachylia ficus	-79.544723	8.931967
Pachylia ficus	-87.240369	20.490749
Pachylia ficus	-76.97997	26.499194
Pachylia ficus	-98.080123	30.203998
Pachylia ficus	-103.501451	25.586535
Pachylia ficus	-92.261667	14.905
Pachylia ficus	-101.234486	19.672356
Pachylia ficus	-102.790554	20.814051
Pachylia ficus	-100.59518	23.548834
Pachylia ficus	-98.254669	19.049351
Pachylia ficus	-99.634651	19.311222
Pachylia ficus	-99.218189	20.006076
Pachylia ficus	-98.24429	18.989021
Pachylia ficus	-104.667756	24.027247
Pachylia ficus	-103.636053	23.639347
Pachylia ficus	-101.926485	21.365372
Pachylia ficus	-102.884024	21.172703
Pachylia ficus	-99.320155	20.068403
Pachylia ficus	-98.750573	20.108426
Pachylia ficus	-102.298156	19.938667
Pachylia ficus	-99.02878	19.261121
Pachylia ficus	-99.048139	19.455137
Pachylia ficus	-84.050864	9.937205
Pachylia ficus	-84.00331	9.930195
Pachylia ficus	-75.596207	6.265964
Pachylia ficus	-75.557189	6.334764
Pachylia ficus	-102.072553	19.425366
Pachylia ficus	-75.617146	6.168535
Pachylia ficus	-99.217676	20.482466
Pachylia ficus	-100.458423	20.678091
Pachylia ficus	-101.527991	20.282233
Pachylia ficus	-69.639984	9.679075
Pachylia ficus	-99.920024	20.375361
Pachylia ficus	-84.287412	9.909808
Pachylia ficus	-99.815436	20.694955
Pachylia ficus	-100.410265	20.593399
Pachylia ficus	-68.254486	10.282892
Pachylia ficus	-93.20254	15.681888
Pachylia ficus	-100.994562	25.43638

Pachylia ficus	-99.132841	19.389228
Pachylia ficus	-99.143333	19.435
Pachylia ficus	-101.429933	20.578919
Pachylia ficus	-99.330136	20.729865
Pachylia ficus	-83.751579	9.784247
Pachylia ficus	-103.281757	20.674175
Pachylia ficus	-84.47123	10.087431
Pachylia ficus	-101.328846	20.71939
Pachylia ficus	-98.36989	26.247452
Pachylia ficus	-99.182108	19.324225
Pachylia ficus	-98.437775	18.921718
Pachylia ficus	-70.361802	9.447771
Pachylia ficus	-98.366497	26.179838
Pachylia ficus	-100.747226	20.91528
Pachylia ficus	-83.650587	9.890422
Pachylia ficus	-99.209167	19.409444
Pachylia ficus	-97.916667	18.566667
Pachylia ficus	-84.797708	10.281891
Pachylia ficus	-75.446509	10.556318
Pachylia ficus	-83.563801	9.831915
Pachylia ficus	-104.902048	21.503038
Pachylia ficus	-101.253105	21.015784
Pachylia ficus	-73.949528	6.324385
Pachylia ficus	-99.376119	23.404859
Pachylia ficus	-73.844204	11.271617
Pachylia ficus	-98.212923	26.245003
Pachylia ficus	-100.995995	22.164181
Pachylia ficus	-96.719895	17.061953
Pachylia ficus	-99.593333	18.96
Pachylia ficus	-80.035605	8.646467
Pachylia ficus	-73.095324	7.105972
Pachylia ficus	-103.319588	20.394757
Pachylia ficus	-103.291005	20.72566
Pachylia ficus	-75.051108	9.157876
Pachylia ficus	-68.8888	12.1404
Pachylia ficus	-98.94542	18.9197
Pachylia ficus	-89.188373	13.707076
Pachylia ficus	-97.479136	18.335828
Pachylia ficus	-99.893333	18.863333
Pachylia ficus	-100.281098	22.419873
Pachylia ficus	-83.680591	8.665542
Pachylia ficus	-92.607778	16.707222
Pachylia ficus	-75.380678	9.315375
Pachylia ficus	-79.535972	8.980625
Pachylia ficus	-70.502617	8.825303
Pachylia ficus	-67.763388	10.489493
Pachylia ficus	-83.589342	8.48008
Pachylia ficus	-87.007861	14.007545

Pachylia ficus	-83.207931	8.786169
Pachylia ficus	-83.458286	8.459236
Pachylia ficus	-84.674735	9.999223
Pachylia ficus	-99.203234	18.783438
Pachylia ficus	-92.134167	16.251111
Pachylia ficus	-97.313276	27.466117
Pachylia ficus	-83.336199	8.404575
Pachylia ficus	-99.256667	18.743333
Pachylia ficus	-92.3837895919	16.4009010666
Pachylia ficus	-86.277639	11.921317
Pachylia ficus	-99.221173	18.921296
Pachylia ficus	-83.201944	8.700764
Pachylia ficus	-84.680702	10.484832
Pachylia ficus	-83.842362	9.237193
Pachylia ficus	-97.388995	26.236704
Pachylia ficus	-83.097542	8.591105
Pachylia ficus	-72.281635	18.448614
Pachylia ficus	-101.155868	19.039153
Pachylia ficus	-85.476473	10.620182
Pachylia ficus	-87.949987	14.772696
Pachylia ficus	-89.047623	16.494101
Pachylia ficus	-80.635347	8.856756
Pachylia ficus	-87.90523	14.871277
Pachylia ficus	-97.102528	18.880369
Pachylia ficus	-83.329421	8.698516
Pachylia ficus	-85.791534	10.432361
Pachylia ficus	-94.613333	16.561667
Pachylia ficus	-103.730833	19.243611
Pachylia ficus	-97.920278	20.264167
Pachylia ficus	-97.100833	18.815
Pachylia ficus	-86.183226	11.905096
Pachylia ficus	-84.27059	10.447984
Pachylia ficus	-85.911804	12.058474
Pachylia ficus	-79.649222	9.077598
Pachylia ficus	-96.1362	15.766019
Pachylia ficus	-61.146168	14.804605
Pachylia ficus	-84.0037	10.431197
Pachylia ficus	-83.50458	10.547196
Pachylia ficus	-86.314393	12.062688
Pachylia ficus	-99.466667	17.36
Pachylia ficus	-70.85671	19.644238
Pachylia ficus	-86.164631	12.023112
Pachylia ficus	-96.916212	19.540076
Pachylia ficus	-61.232596	10.699848
Pachylia ficus	-93.302513	16.245372
Pachylia ficus	-61.458717	10.712525
Pachylia ficus	-101.520082	17.649243

Pachylia ficus	-107.385816	24.823739
Pachylia ficus	-95.056389	18.472222
Pachylia ficus	-93.033333	16.733333
Pachylia ficus	-95.348333	18.455
Pachylia ficus	-61.11768	14.7288
Pachylia ficus	-69.916874	18.460162
Pachylia ficus	-92.93238	18.006228
Pachylia ficus	-104.964431	19.390433
Pachylia ficus	-82.705401	27.495836
Pachylia ficus	-66.021204	18.18636
Pachylia ficus	-89.633478	21.018412
Pachylia ficus	-82.500216	27.344489
Pachylia ficus	-97.979589	16.42926
Pachylia ficus	-88.978421	17.099867
Pachylia ficus	-61.05748	14.70243
Pachylia ficus	-82.563903	27.385722
Pachylia ficus	-90.887086	16.82257
Pachylia ficus	-93.116718	16.73549
Pachylia ficus	-70.462529	19.776087
Pachylia ficus	-82.530986	27.299357
Pachylia ficus	-91.331667	16.216667
Pachylia ficus	-94.357946	18.111388
Pachylia ficus	-74.098804	18.255401
Pachylia ficus	-99.859722	16.829167
Pachylia ficus	-91.063611	16.707222
Pachylia ficus	-88.006011	15.397303
Pachylia ficus	-88.397204	18.66205
Pachylia ficus	-61.04794	14.78458
Pachylia ficus	-88.779275	17.258536
Pachylia ficus	-94.497338	18.143545
Pachylia ficus	-95.104202	18.427151
Pachylia ficus	-95.201485	18.445438
Pachylia ficus	-66.111805	18.404521
Pachylia ficus	-61.522999	16.229015
Pachylia ficus	-81.656137	26.715734
Pachylia ficus	-61.06584	14.51928
Pachylia ficus	-66.063842	18.455542
Pachylia ficus	-95.066667	18.583333
Pachylia ficus	-87.431131	20.215574
Pachylia ficus	-60.89725	14.5173
Pachylia ficus	-77.399833	18.465884
Pachylia ficus	-87.599722	20.885556
Pachylia ficus	-61.06954	14.63975
Pachylia ficus	-77.164742	18.429997
Pachylia ficus	-96.308899	18.112439
Pachylia ficus	-86.840374	21.137628
Pachylia ficus	-60.91224	14.57353
Pachylia ficus	-61.01367	14.51401

Pachylia ficus	-87.092178	20.604103
Pachylia ficus	-68.379565	18.516018
Pachylia ficus	-61.14822	14.65077
Pachylia ficus	-61.770735	16.093956
Pachylia ficus	-61.21694	14.835
Pachylia ficus	-81.819627	26.611568
Pachylia ficus	-106.410465	23.23536
Pachylia ficus	-86.823966	21.201395
Pachylia ficus	-86.968853	20.739626
Pachylia ficus	-60.89986	14.46382
Pachylia ficus	-63.021634	18.043441
Pachylia ficus	-105.228333	20.62
Pachylia ficus	-82.058283	26.433826
Pachylia ficus	-64.96322	18.334224
Pachylia ficus	-81.933845	26.49578
Pachylia ficus	-81.781295	26.448978
Pachylia ficus	-81.922666	26.552364
Pachylia ficus	-62.734087	17.292362
Pachylia ficus	-97.434417	20.947008
Pachylia ficus	-81.363372	25.957067
Pachylia ficus	-80.223625	27.200455
Pachylia ficus	-98.386744	21.158032
Pachylia ficus	-80.308647	25.614388
Pachylia ficus	-80.358187	25.62932
Pachylia ficus	-80.583942	25.395437
Pachylia ficus	-80.281415	25.661768
Pachylia ficus	-80.28345	25.311972
Pachylia ficus	-80.433321	25.692914
Pachylia ficus	-80.265106	26.694758
Pachylia ficus	-80.42482	25.749263
Pachylia ficus	-80.36949	25.176262
Pachylia ficus	-80.144256	26.380599
Pachylia ficus	-80.276378	25.718951
Pachylia ficus	-80.37365	25.759819
Pachylia ficus	-80.070283	26.3661
Pachylia ficus	-80.179813	25.823983
Pachylia ficus	-80.089541	26.809227
Pachylia ficus	-80.240069	26.258168
Pachylia ficus	-80.323496	25.730497
Pachylia ficus	-80.061447	26.618502
Pachylia ficus	-80.287911	26.212983
Pachylia ficus	-80.174508	25.685358
Pachylia ficus	-80.189972	25.777008
Pachylia ficus	-80.237222	26.086957
Pachylia ficus	-80.150214	26.163753
Pachylia ficus	-80.214061	26.152598
Pachylia ficus	-80.287393	25.820613
Pachylia ficus	-81.756645	24.565435

Pachylia ficus	-81.805548	24.559836
Pachylia ficus	-81.404975	24.658178
