# Supplementary material for: Future distribution of the epiphytic leafless orchid (Dendrophylax lindenii), its pollinators and phorophytes evaluated using niche modelling and three different climate change projections
Source: Sci Rep. 2023 Sep 14;13:15242. doi: 10.1038/s41598-023-42573-5 (PMC10502118; doi:10.1038/s41598-023-42573-5)
Supplement: Supplementary file 2 — Supplementary Information 2. [file 41598_2023_42573_MOESM2_ESM.pdf]

Future of epiphytic, leafless orchid (*Dendrophylax lindenii*) – complex modelling of the orchid, its pollinators and phorophytes

Marta Kolanowska[a*]

[a] University of Lodz, Faculty of Biology and Environmental Protection, Department of Geobotany and Plant Ecology, Banacha 12/16, 90-237 Lodz, Poland

* Corresponding author

Supplementary Annex 2. Results of Pearson's Correlation Coefficient (R) statistics.

	bio1	bio2	bio3	bio4	bio5	bio6	bio7	bio8	bio9	bio10	bio11	bio12	bio13	bio14	bio15	bio16	bio17	bio18	bio19
bio1	x	0.894	0.974	0.653	0.983	0.897	0.834	0.991	0.991	0.991	0.982	0.85	0.877	0.622	0.894	0.874	0.651	0.85	0.639
bio2		x	0.897	0.835	0.955	0.622	0.976	0.927	0.863	0.93	0.816	0.647	0.706	0.462	0.945	0.702	0.481	0.749	0.421
bio3			x	0.582	0.957	0.881	0.808	0.958	0.974	0.956	0.97	0.858	0.889	0.597	0.909	0.887	0.634	0.857	0.637
bio4				x	0.768	0.271	0.934	0.736	0.58	0.746	0.499	0.363	0.391	0.405	0.682	0.382	0.396	0.524	0.206
bio5					x	0.803	0.92	0.994	0.962	0.997	0.935	0.788	0.825	0.589	0.925	0.821	0.613	0.827	0.573
bio6						x	0.507	0.84	0.925	0.835	0.959	0.884	0.889	0.603	0.701	0.889	0.643	0.777	0.728
bio7							x	0.888	0.785	0.895	0.723	0.562	0.612	0.457	0.881	0.606	0.465	0.689	0.353
bio8								x	0.973	0.998	0.952	0.812	0.841	0.615	0.906	0.837	0.639	0.844	0.595
bio9									x	0.972	0.991	0.867	0.895	0.614	0.885	0.893	0.647	0.847	0.668
bio10										x	0.949	0.809	0.838	0.616	0.904	0.834	0.64	0.836	0.598
bio11											x	0.882	0.906	0.61	0.854	0.905	0.646	0.841	0.681
bio12												x	0.975	0.795	0.669	0.977	0.832	0.901	0.823
bio13													x	0.687	0.767	0.997	0.725	0.902	0.771
bio14														x	0.347	0.686	0.984	0.764	0.632
bio15															x	0.765	0.374	0.747	0.433
bio16																x	0.723	0.9	0.777
bio17																	x	0.784	0.686
bio18																		x	0.609
bio19																			x
