# Supplementary material for: Future distribution of the epiphytic leafless orchid (Dendrophylax lindenii), its pollinators and phorophytes evaluated using niche modelling and three different climate change projections
Source: Sci Rep. 2023 Sep 14;13:15242. doi: 10.1038/s41598-023-42573-5 (PMC10502118; doi:10.1038/s41598-023-42573-5)
Supplement: Supplementary file 5 — Supplementary Information 5. [file 41598_2023_42573_MOESM5_ESM.pdf]

Future of epiphytic, leafless orchid (*Dendrophylax lindenii*) – complex modelling of the orchid, its pollinators and phorophytes

Marta Kolanowska[a*]

[a] University of Lodz, Faculty of Biology and Environmental Protection, Department of Geobotany and Plant Ecology, Banacha 12/16, 90-237 Lodz, Poland

* Corresponding author

Supplementary Annex 5. Changes in the coverage (km^2) of suitable niches of pollinators and phorophytes of *D. lindenii*.

Species	Projection	Scenario	Range expansion	Permanent range	Range contraction	Change
Dendrophylax lindenii	CNRM	SSP1-2.6	353.0521	24941.54	3893.069	-12.28%
		SSP2-4.5	3912.923	25914.37	2920.233	+3.44%
		SSP3-7.0	1509.751	15414.31	13420.3	-41.31%
		SSP5-8.5	2215.855	5956.136	22878.47	-71.66%
	GISS	SSP1-2.6	1729.006	26092.19	2742.412	-3.51%
		SSP2-4.5	1168.784	25413.71	3420.894	-7.81%
		SSP3-7.0	3157.615	24397.72	4436.89	-4.44%
		SSP5-8.5	835.5855	22771.43	6063.174	-18.13%
	INM	SSP1-2.6	7608.317	28354.66	479.9437	+24.72%
		SSP2-4.5	8695.959	28767.28	67.33024	+29.92%
		SSP3-7.0	1426.02	24837.09	3997.517	-8.92%
		SSP5-8.5	171.7784	21182.27	7652.34	-25.94%
Dolba hyloeus	CNRM	SSP1-2.6	25005.41	114232.7	18.12737	+21.87%
		SSP2-4.5	28733.61	114174	76.82553	+25.08%
		SSP3-7.0	10775.43	103331.2	10919.58	-0.13%
		SSP5-8.5	8348.949	95018.5	19232.28	-9.53%
	GISS	SSP1-2.6	12690.89	110053	4197.781	+7.43%
		SSP2-4.5	20582.34	114174	76.82553	+17.95%
		SSP3-7.0	28278.7	114245.6	5.179249	+24.75%
		SSP5-8.5	32265.86	114249.1	1.726416	+28.24%
	INM	SSP1-2.6	11621.37	108506.1	5744.65	+5.14%
		SSP2-4.5	16192.92	114248.2	2.589625	+14.17%
		SSP3-7.0	24641.14	114250.8	0	+21.57%
		SSP5-8.5	27292.92	114250.8	0	+23.89%
Cocytius antaeus	CNRM	SSP1-2.6	0	291887.8	10399.93	-3.44%
		SSP2-4.5	0	250601.4	51686.32	-17.10%

		SSP3-7.0	0	240319.7	61967.99	-20.50%
		SSP5-8.5	0	223137.6	79150.15	-26.18%
	GISS	SSP1-2.6	0	298139.2	4148.578	-1.37%
		SSP2-4.5	0	271350.4	30937.38	-10.23%
		SSP3-7.0	0	260806.3	41481.47	-13.72%
		SSP5-8.5	0	249197.8	53089.89	-17.56%
	INM	SSP1-2.6	0	296670	5617.759	-1.86%
		SSP2-4.5	101.8586	301567.8	719.9156	-0.20%
		SSP3-7.0	0	280937.1	21350.59	-7.06%
		SSP5-8.5	0	285582.1	16705.67	-5.53%
Pachylia ficus	CNRM	SSP1-2.6	2142.483	328682	1131.666	+0.31%
		SSP2-4.5	3962.989	324007.8	5805.938	-0.56%
		SSP3-7.0	6970.406	320593.8	9219.926	-0.68%
		SSP5-8.5	1773.893	265257.8	64555.89	-19.04%
	GISS	SSP1-2.6	10380.94	329316.5	497.2079	+3.00%
		SSP2-4.5	1965.525	329105.9	707.8307	+0.38%
		SSP3-7.0	5757.599	326967.7	2845.997	+0.88%
		SSP5-8.5	350.4625	321037.5	8776.237	-2.55%
	INM	SSP1-2.6	2065.657	329501.2	312.4814	+0.53%
		SSP2-4.5	14545.06	329465.8	347.8729	+4.30%
		SSP3-7.0	14577.86	328394.6	1419.114	+3.99%
		SSP5-8.5	13489.35	325188.6	4625.069	+2.69%
Annona glabra	CNRM	SSP1-2.6	341.8304	320603.3	4211.593	-1.19%
		SSP2-4.5	2124.355	322708.6	2106.228	+0.01%
		SSP3-7.0	7537.534	324226.2	588.708	+2.14%
		SSP5-8.5	2142.483	308050.5	16764.37	-4.50%
	GISS	SSP1-2.6	3274.149	324796.7	18.12737	+1.00%
		SSP2-4.5	1896.468	323761.8	1053.114	+0.26%
		SSP3-7.0	9466.804	324631.9	183.0001	+2.86%

		SSP5-8.5	6600.953	323950.8	864.0714	+1.77%
	INM	SSP1-2.6	6707.991	324812.3	2.589625	+2.06%
		SSP2-4.5	24874.21	324737.2	77.68874	+7.63%
		SSP3-7.0	19114.88	324154.5	660.3543	+5.68%
		SSP5-8.5	15664.64	324448.9	366.0003	+4.71%
Comocladia dentata	CNRM	SSP1-2.6	4719.159	171845.8	10601.92	-3.22%
		SSP2-4.5	8628.629	155067.6	27380.1	-10.28%
		SSP3-7.0	10027.03	165545.2	16902.48	-3.77%
		SSP5-8.5	5100.697	129918.9	52528.81	-26.00%
	GISS	SSP1-2.6	7644.572	181814.9	632.7316	+3.84%
		SSP2-4.5	7648.024	180677.2	1770.44	+3.22%
		SSP3-7.0	8042.511	182180.1	267.5945	+4.26%
		SSP5-8.5	7732.619	181496.4	951.2554	+3.72%
	INM	SSP1-2.6	2961.667	171603.2	10844.48	-4.32%
		SSP2-4.5	10151.33	178663.4	3784.305	+3.49%
		SSP3-7.0	6902.213	181321.2	1126.487	+3.17%
		SSP5-8.5	13643.87	180451.1	1996.601	+6.38%
Diospyros crassinervis	CNRM	SSP1-2.6	13196.73	184218.1	17591.32	-2.18%
		SSP2-4.5	14898.11	148747.2	53062.27	-18.91%
		SSP3-7.0	11447.87	84603.9	117205.5	-52.40%
		SSP5-8.5	11680.07	16884.35	184925.1	-85.85%
	GISS	SSP1-2.6	4005.286	179385.9	22423.56	-9.13%
		SSP2-4.5	5534.891	160413.4	41396.01	-17.77%
		SSP3-7.0	9571.252	109385.7	92423.7	-41.05%
		SSP5-8.5	9040.379	139776.7	62032.73	-26.26%
	INM	SSP1-2.6	1394.944	178962.9	22846.53	-10.63%
		SSP2-4.5	6970.406	171318.3	30491.1	-11.65%
		SSP3-7.0	11016.26	163328.5	38480.96	-13.61%
		SSP5-8.5	11942.49	156567	45242.47	-16.50%

Erythroxylum areolatum	CNRM	SSP1-2.6	22011.81	264220.3	0	+8.33%
		SSP2-4.5	29967.13	264220.3	0	+11.34%
		SSP3-7.0	35564.18	264220.3	0	+13.46%
		SSP5-8.5	42480.2	264220.3	0	+16.08%
	GISS	SSP1-2.6	19262.49	264220.3	0	+7.29%
		SSP2-4.5	23776.21	264220.3	0	+9.00%
		SSP3-7.0	28648.15	264220.3	0	+10.84%
		SSP5-8.5	28483.28	264206.4	13.81133	+10.77%
	INM	SSP1-2.6	9084.403	264030.3	189.9058	+3.37%
		SSP2-4.5	21227.15	264214.2	6.042457	+8.03%
		SSP3-7.0	29344.76	264220.3	0	+11.11%
		SSP5-8.5	33397.52	264220.3	0	+12.64%
Fraxinus caroliniana	CNRM	SSP1-2.6	9.49529	141034.4	1564.133	-1.09%
		SSP2-4.5	88.91044	138432.7	4165.843	-2.86%
		SSP3-7.0	0	115089.8	27508.72	-19.29%
		SSP5-8.5	0	45889.01	96709.53	-67.82%
	GISS	SSP1-2.6	190.769	141277.8	1320.709	-0.79%
		SSP2-4.5	150.1982	141262.3	1336.246	-0.83%
		SSP3-7.0	15.53775	134145.1	8453.398	-5.92%
		SSP5-8.5	0	116405.3	26193.19	-18.37%
	INM	SSP1-2.6	7.768874	141741.4	857.1657	-0.60%
		SSP2-4.5	88.91044	140933.4	1665.129	-1.11%
		SSP3-7.0	69.91986	132629.3	9969.191	-6.94%
		SSP5-8.5	21.5802	133001.4	9597.148	-6.72%
