# Supplementary material for: Future distribution of the epiphytic leafless orchid (Dendrophylax lindenii), its pollinators and phorophytes evaluated using niche modelling and three different climate change projections
Source: Sci Rep. 2023 Sep 14;13:15242. doi: 10.1038/s41598-023-42573-5 (PMC10502118; doi:10.1038/s41598-023-42573-5)
Supplement: Supplementary file 7 — Supplementary Information 7. [file 41598_2023_42573_MOESM7_ESM.pdf]

Future of epiphytic, leafless orchid (*Dendrophylax lindenii*) – complex modelling of the orchid, its pollinators and phorophytes

Marta Kolanowska[a*]

[a] University of Lodz, Faculty of Biology and Environmental Protection, Department of Geobotany and Plant Ecology, Banacha 12/16, 90-237 Lodz, Poland

* Corresponding author

Supplementary Annex 7. Predicted changes in the distribution of *D. lindenii* phorophytes. Maps created in ArcGIS using MaxEnt results.

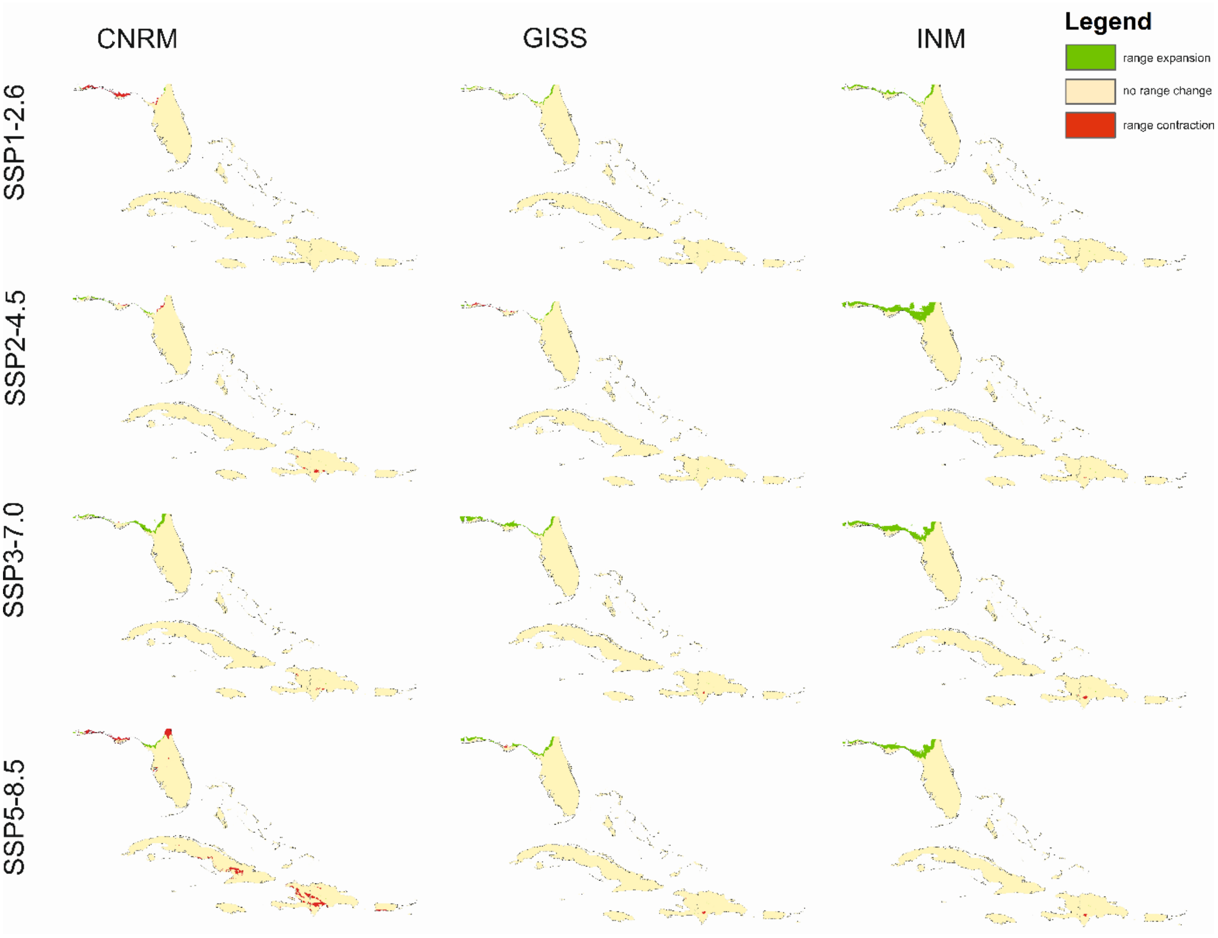

Fig. 1. Changes in the distribution of suitable niches of *Annona glabra* in various climate change scenarios.

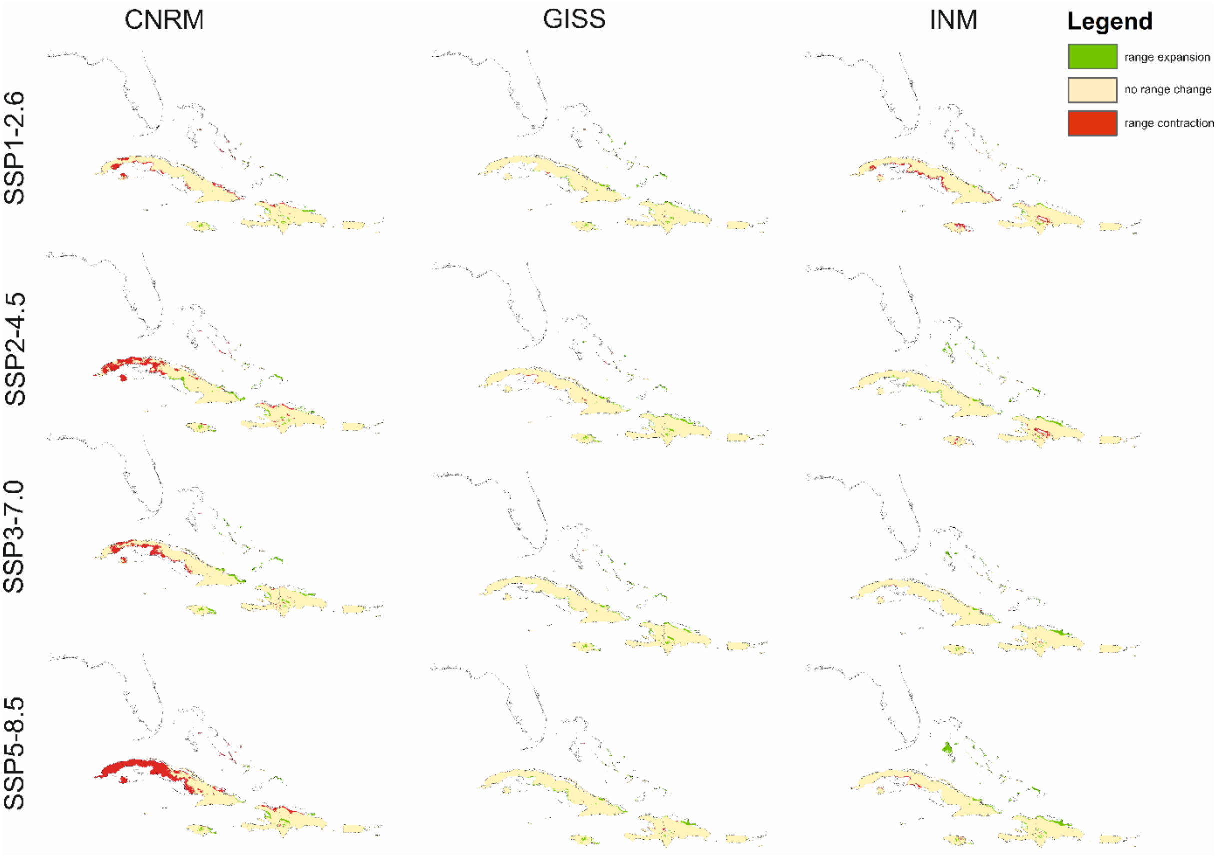

Fig. 2. Changes in the distribution of suitable niches of *Comocladia dentata* in various climate change scenarios.

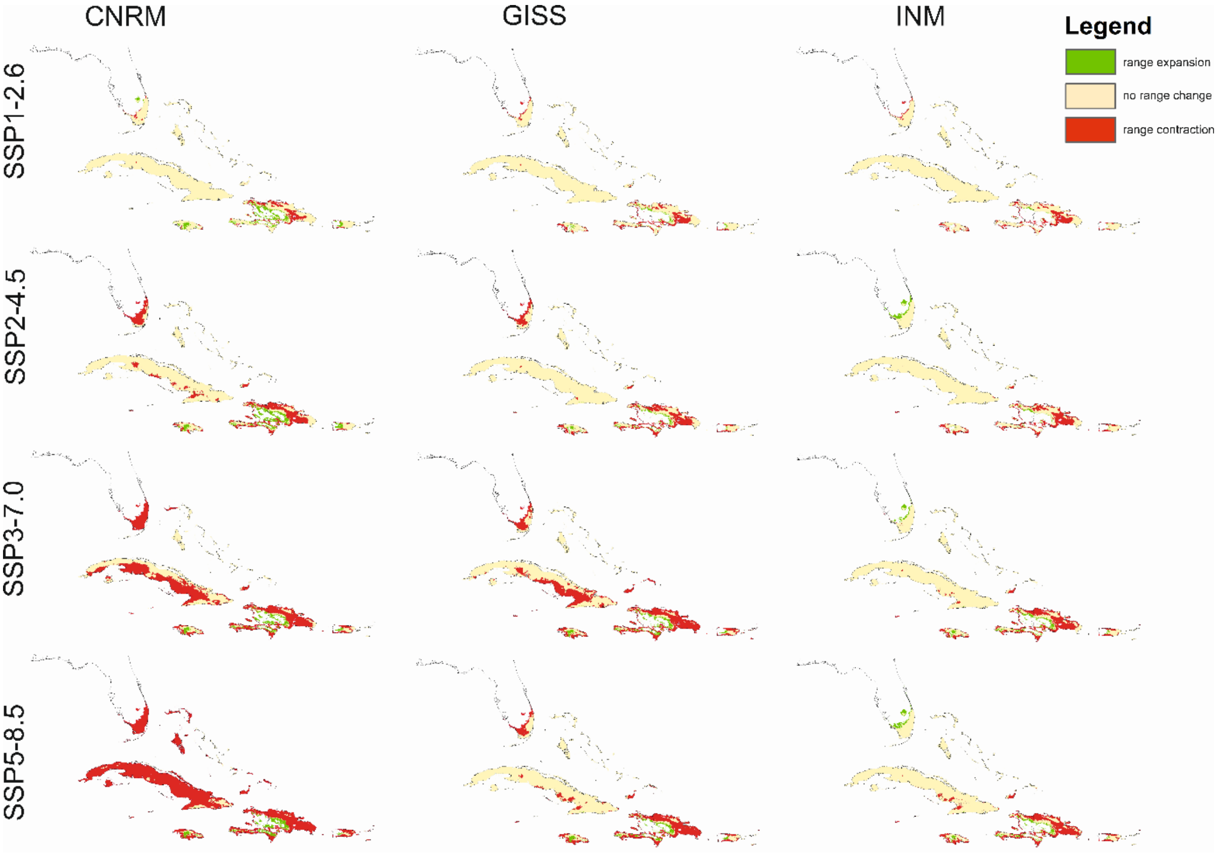

Fig. 3. Changes in the distribution of suitable niches of *Diospyros crassinervis* in various climate change scenarios.

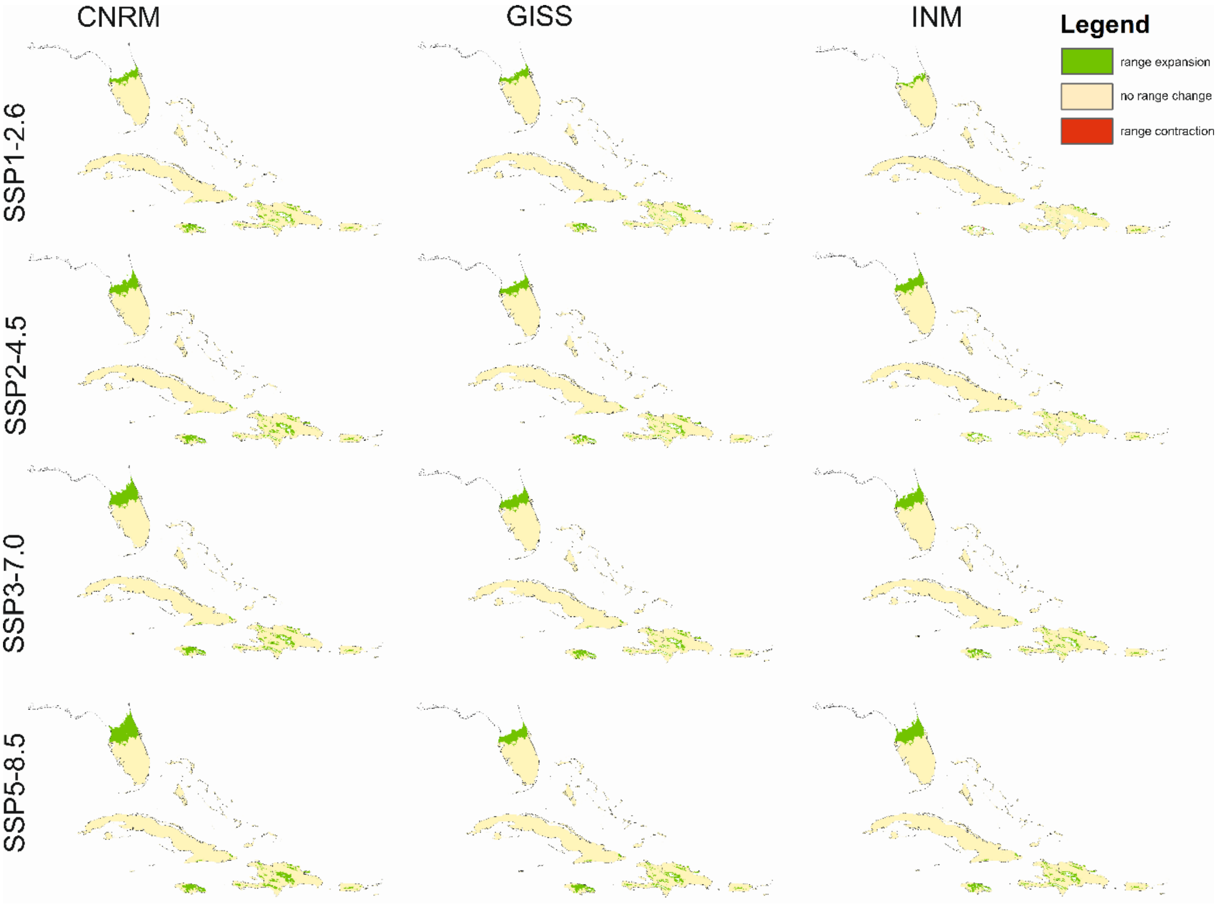

Fig. 4. Changes in the distribution of suitable niches of *Erythroxylum areolatum* in various climate change scenarios.

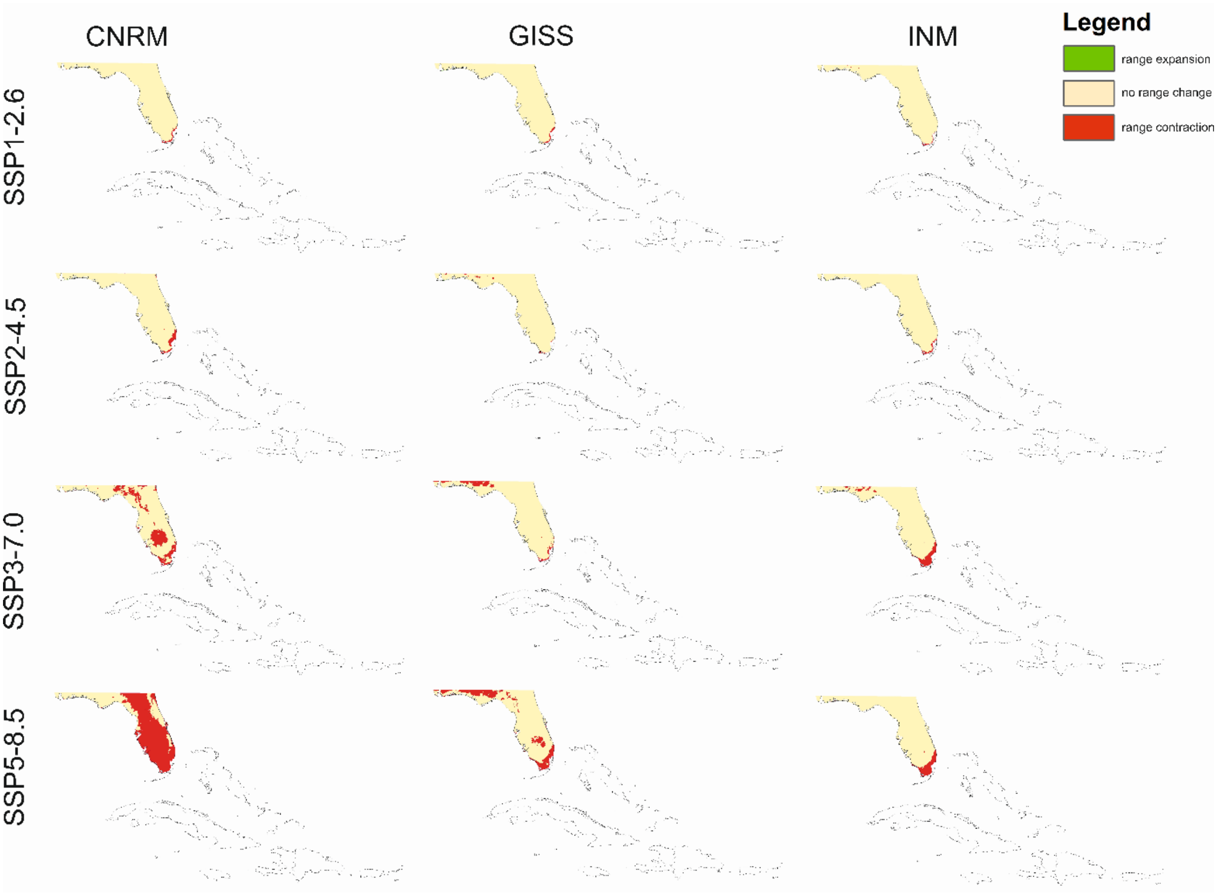

Fig. 5. Changes in the distribution of suitable niches of *Fraxinus caroliniana* in various climate change scenarios.
