# Supplementary material for: Future distribution of the epiphytic leafless orchid (Dendrophylax lindenii), its pollinators and phorophytes evaluated using niche modelling and three different climate change projections
Source: Sci Rep. 2023 Sep 14;13:15242. doi: 10.1038/s41598-023-42573-5 (PMC10502118; doi:10.1038/s41598-023-42573-5)
Supplement: Supplementary file 8 — Supplementary Information 8. [file 41598_2023_42573_MOESM8_ESM.pdf]

Future of epiphytic, leafless orchid (*Dendrophylax lindenii*) – complex modelling of the orchid, its pollinators and phorophytes

Marta Kolanowska[a*]

[a] University of Lodz, Faculty of Biology and Environmental Protection, Department of Geobotany and Plant Ecology, Banacha 12/16, 90-237 Lodz, Poland

* Corresponding author

Supplementary Annex 8. Overlap between suitable niches of *D. lindenii*, its pollinators and phorophytes. Maps created in ArcGIS using MaxEnt results.

Species	Projection	Scenario	Overlap with potential range of *D. lindenii*
Dolba hyloeus	CNRM	SSP1-2.6	84.94%
		SSP2-4.5	89.16%
		SSP3-7.0	54.59%
		SSP5-8.5	30.01%
	GISS	SSP1-2.6	60.96%
		SSP2-4.5	76.76%
		SSP3-7.0	82.38%
		SSP5-8.5	98.43%
	INM	SSP1-2.6	59.08%
		SSP2-4.5	65.93%
		SSP3-7.0	81.23%
		SSP5-8.5	93.01%
	PRESENT		24,83%
Cocytius antaeus	CNRM	SSP1-2.6	100.00%
		SSP2-4.5	71.82%
		SSP3-7.0	50.24%
		SSP5-8.5	26.93%
	GISS	SSP1-2.6	100.00%
		SSP2-4.5	99.99%
		SSP3-7.0	97.69%
		SSP5-8.5	84.93%
	INM	SSP1-2.6	100.00%
		SSP2-4.5	100.00%
		SSP3-7.0	100.00%

		SSP5-8.5	100.00%
	PRESENT		100,00%
Pachylia ficus	CNRM	SSP1-2.6	100.00%
		SSP2-4.5	100.00%
		SSP3-7.0	100.00%
		SSP5-8.5	99.72%
	GISS	SSP1-2.6	100.00%
		SSP2-4.5	100.00%
		SSP3-7.0	100.00%
		SSP5-8.5	100.00%
	INM	SSP1-2.6	100.00%
		SSP2-4.5	100.00%
		SSP3-7.0	100.00%
		SSP5-8.5	100.00%
	PRESENT		100,00%
Annona glabra	CNRM	SSP1-2.6	100.00%
		SSP2-4.5	100.00%
		SSP3-7.0	100.00%
		SSP5-8.5	100.00%
	GISS	SSP1-2.6	100.00%
		SSP2-4.5	100.00%
		SSP3-7.0	100.00%
		SSP5-8.5	100.00%
	INM	SSP1-2.6	100.00%
		SSP2-4.5	100.00%
		SSP3-7.0	100.00%
		SSP5-8.5	100.00%
	PRESENT		100,00%
Comocladia dentata	CNRM	SSP1-2.6	0.01%

		SSP2-4.5	6.16%
		SSP3-7.0	8.80%
		SSP5-8.5	6.67%
	GISS	SSP1-2.6	0.00%
		SSP2-4.5	1.19%
		SSP3-7.0	7.53%
		SSP5-8.5	0.65%
	INM	SSP1-2.6	0.23%
		SSP2-4.5	0.00%
		SSP3-7.0	3.05%
		SSP5-8.5	0.00%
	PRESENT		0,08%
Diospyros crassinervis	CNRM	SSP1-2.6	31.02%
		SSP2-4.5	10.82%
		SSP3-7.0	6.97%
		SSP5-8.5	0.04%
	GISS	SSP1-2.6	24.01%
		SSP2-4.5	15.15%
		SSP3-7.0	18.56%
		SSP5-8.5	13.94%
	INM	SSP1-2.6	25.91%
		SSP2-4.5	37.62%
		SSP3-7.0	44.38%
		SSP5-8.5	46.33%
	PRESENT		31,56%
Erythroxylum areolatum	CNRM	SSP1-2.6	100.00%
		SSP2-4.5	100.00%
		SSP3-7.0	100.00%
		SSP5-8.5	100.00%

	GISS	SSP1-2.6	100.00%
		SSP2-4.5	100.00%
		SSP3-7.0	100.00%
		SSP5-8.5	100.00%
	INM	SSP1-2.6	100.00%
		SSP2-4.5	100.00%
		SSP3-7.0	100.00%
		SSP5-8.5	100.00%
	PRESENT		100,00%
Fraxinus caroliniana	CNRM	SSP1-2.6	95.30%
		SSP2-4.5	87.23%
		SSP3-7.0	70.12%
		SSP5-8.5	0.54%
	GISS	SSP1-2.6	97.12%
		SSP2-4.5	94.32%
		SSP3-7.0	84.03%
		SSP5-8.5	66.35%
	INM	SSP1-2.6	96.11%
		SSP2-4.5	96.00%
		SSP3-7.0	73.80%
		SSP5-8.5	77.20%
	PRESENT		98.59%
