# Supplementary material for: Future distribution of the epiphytic leafless orchid (Dendrophylax lindenii), its pollinators and phorophytes evaluated using niche modelling and three different climate change projections
Source: Sci Rep. 2023 Sep 14;13:15242. doi: 10.1038/s41598-023-42573-5 (PMC10502118; doi:10.1038/s41598-023-42573-5)
Supplement: Supplementary file 9 — Supplementary Information 9. [file 41598_2023_42573_MOESM9_ESM.pdf]

Future of epiphytic, leafless orchid (*Dendrophylax lindenii*) – complex modelling of the orchid, its pollinators and phorophytes

Marta Kolanowska[a*]

[a] University of Lodz, Faculty of Biology and Environmental Protection, Department of Geobotany and Plant Ecology, Banacha 12/16, 90-237 Lodz, Poland

* Corresponding author

Supplementary Annex 9. Predicted future overlaps between suitable niches for *D. lindenii* (hatched red) and those of its pollinators (grey). Maps created in ArcGIS using MaxEnt results.

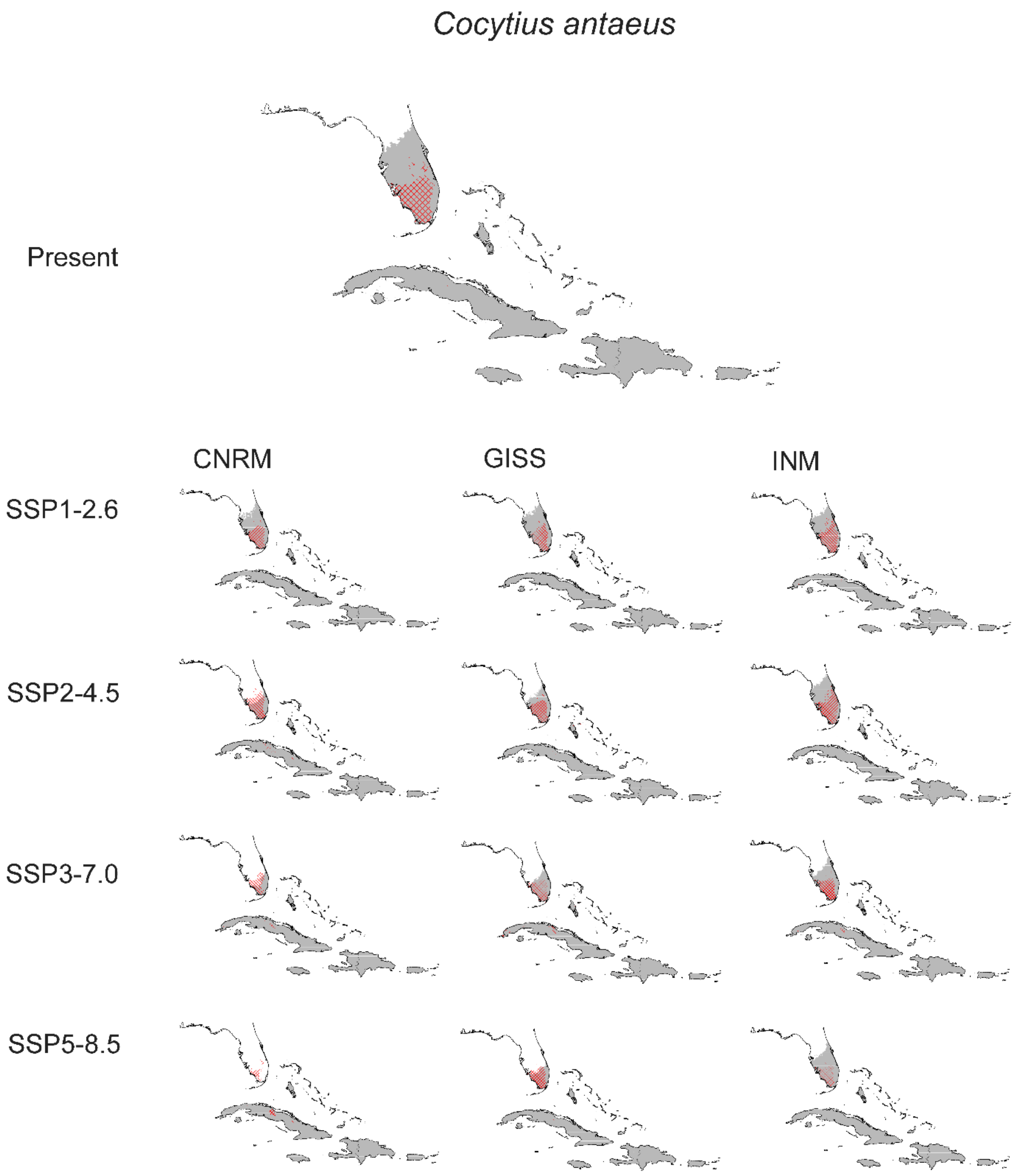

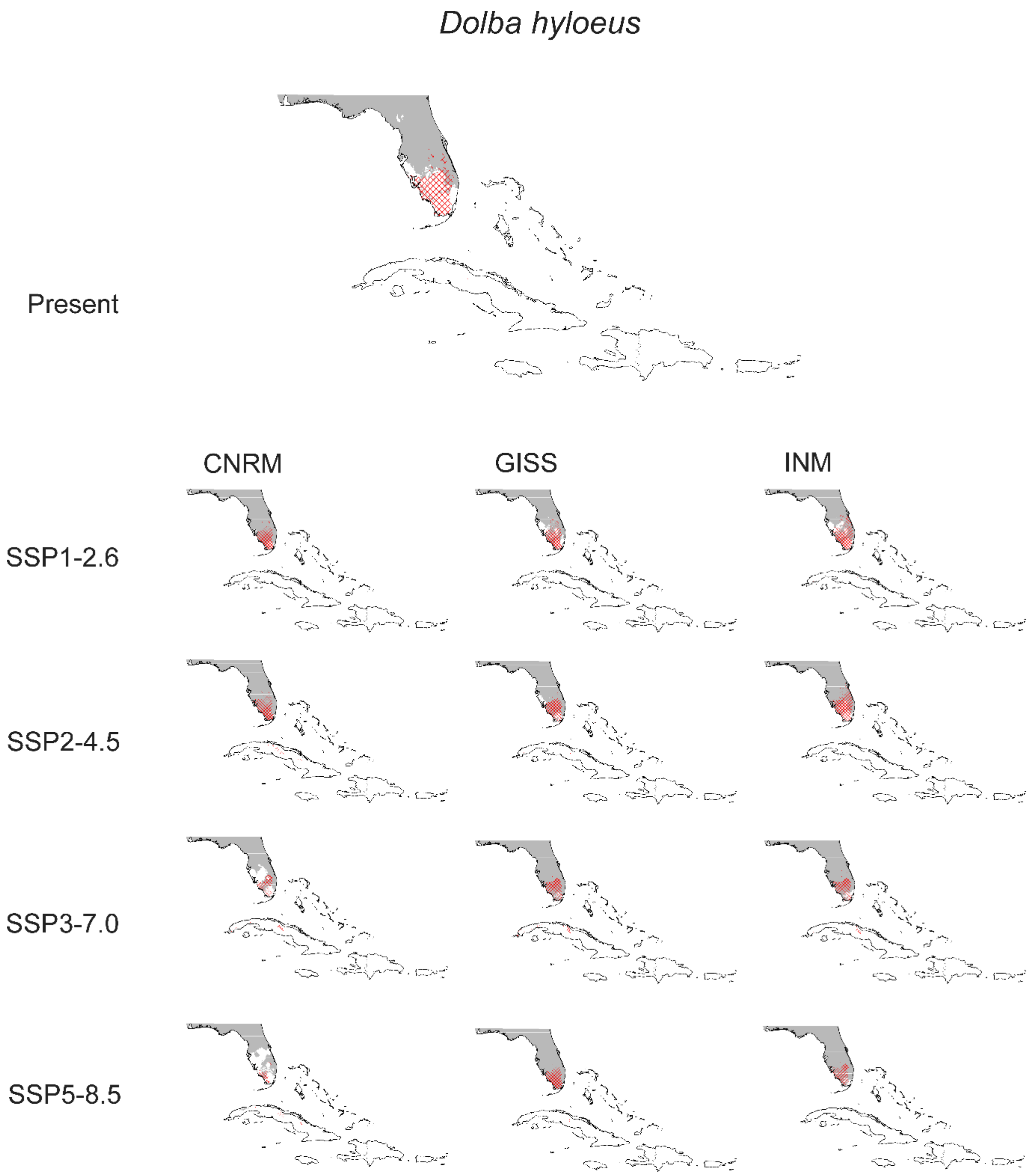
Dolba hyloeus
Present
CNRM
GISS
INM
SSP1-2.6
SSP2-4.5
SSP3-7.0
SSP5-8.5

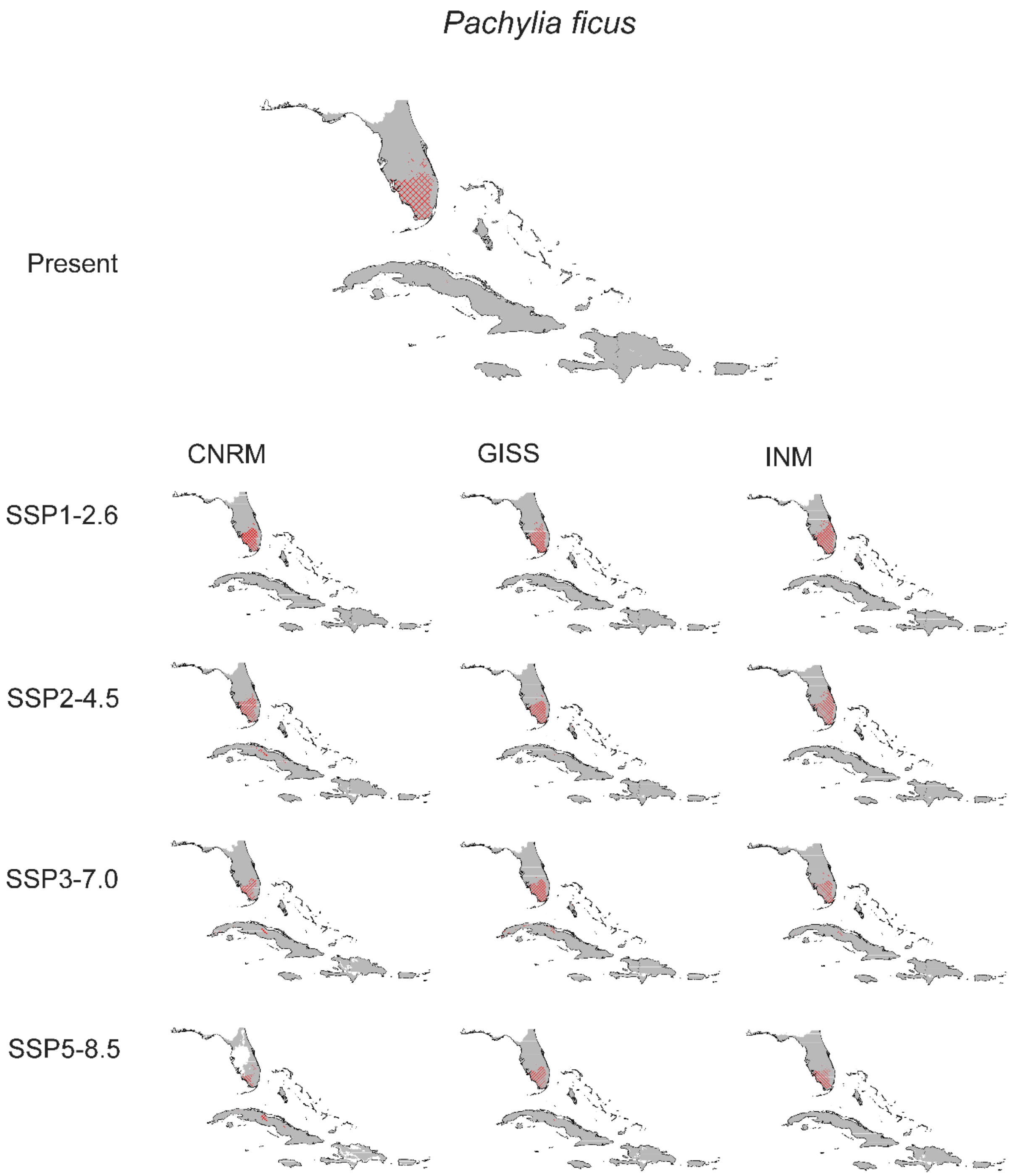
Pachylia ficus
Present
CNRM
GISS
INM
SSP1-2.6
SSP2-4.5
SSP3-7.0
SSP5-8.5
