# Supplementary material for: Future distribution of the epiphytic leafless orchid (Dendrophylax lindenii), its pollinators and phorophytes evaluated using niche modelling and three different climate change projections
Source: Sci Rep. 2023 Sep 14;13:15242. doi: 10.1038/s41598-023-42573-5 (PMC10502118; doi:10.1038/s41598-023-42573-5)

**Future of epiphytic, leafless orchid (*Dendrophylax lindenii*) – complex modelling of the orchid, its pollinators and phorophytes**

**Marta Kolanowska**[a*]

[a] University of Lodz, Faculty of Biology and Environmental Protection, Department of Geobotany and Plant Ecology, Banacha 12/16, 90-237 Lodz, Poland

* Corresponding author

**Supplementary Annex 11**. Observations of flowering *D. lindenii* and adult individuals of orchid pollinators. Graph generated using public data available in iNaturalist.

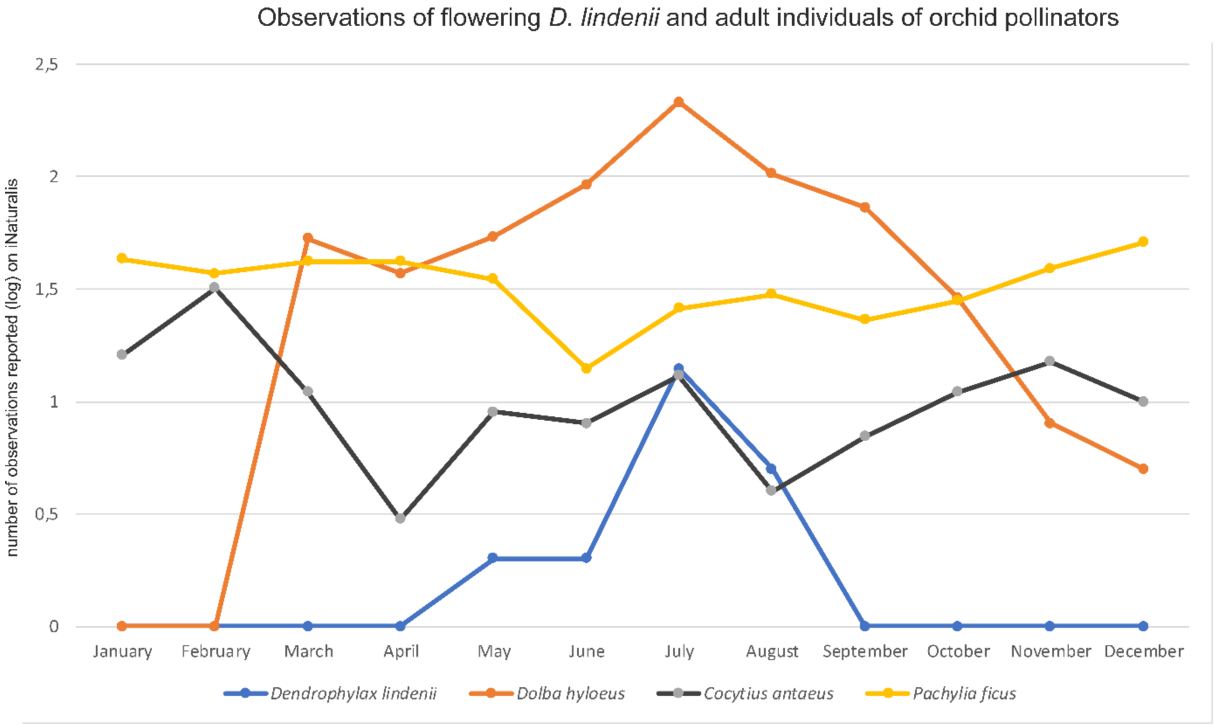

Supplement: Supplementary file 11 — Supplementary Information 11. [file 41598_2023_42573_MOESM11_ESM.pdf]
